# Proteomic Analysis of Mamestra Brassicae Nucleopolyhedrovirus Progeny Virions from Two Different Hosts

**DOI:** 10.1371/journal.pone.0153365

**Published:** 2016-04-08

**Authors:** Dianhai Hou, Xi Chen, Lei-Ke Zhang

**Affiliations:** 1 State Key Laboratory of Virology, Wuhan Institute of Virology, Chinese Academy of Sciences, Wuhan, 430071, China; 2 Wuhan Institute of Biotechnology, Wuhan, P. R. China; Ecole des Mines d'Alès, FRANCE

## Abstract

Mamestra brassicae nucleopolyhedrovirus (MabrNPV) has a wide host range replication in more than one insect species. In this study, a sequenced MabrNPV strain, MabrNPV-CTa, was used to perform proteomic analysis of both BVs and ODVs derived from two infected hosts: *Helicoverpa armigera* and *Spodoptera exigua*. A total of 82 and 39 viral proteins were identified in ODVs and BVs, respectively. And totally, 23 and 76 host proteins were identified as virion-associated with ODVs and BVs, respectively. The host proteins incorporated into the virus particles were mainly involved in cytoskeleton, signaling, vesicle trafficking, chaperone and metabolic systems. Some host proteins, such as actin, cyclophilin A and heat shock protein 70 would be important for viral replication. Several host proteins involved in immune response were also identified in BV, and a C-type lectin protein was firstly found to be associated with BV and its family members have been demonstrated to be involved in entry process of other viruses. This study facilitated the annotation of baculovirus genome, and would help us to understand baculovirus virion structure. Furthermore, the identification of host proteins associated with virions produced *in vivo* would facilitate investigations on the involvement of intriguing host proteins in virus replication.

## Introduction

Baculoviruses are a family of enveloped rod-shaped viruses with large double-stranded, circular DNA genome. Baculoviruses are generally host specific, mainly infecting insects in the orders *Lepidoptera*, *Hymenoptera*, and *Diptera*. Correspondingly, based on phylogeny, the family *Baculoviridae* can be divided into four genera: *Alphabaculovirus* [lepidopteran nucleopolyhedrovirus (NPV)], *Betabaculovirus* [lepidopteran granulovirus (GV)], *Gammabaculovirus* (hymenopteran NPV) and *Deltabaculovirus* (dipteran NPV). The alphabaculoviruses can be further divided into two subgroups: Group I and Group II NPVs [1].

The lepidopteran baculoviruses are characterized by a unique biphasic replication cycle in which two progeny phenotypes are produced: the budded virus (BV) and the occlusion-derived virus (ODV). ODVs are embedded in occlusion bodies (OBs), which can benefit the survival of virus in the environment, therefore contributing to its dissemination. Once ingested by a susceptible insect, ODVs are released from OBs within the larval midgut and initiate oral infection. At the early stage of the infection, nucleocapsid assembly is accomplished in the nuclei of infected host cells, and some nucleocapsids are transported through the nuclear membranes to the cytoplasm and finally budded out of the cytoplasmic membrane to produce infectious BVs. The produced BVs in midgut cells disseminate infection among cells and tissues to establish systematic infection *in vivo*, and BVs are also responsible for *in vitro* infection in cell cultures [2, 3]. While some the retaining nucleocapsids become enveloped in the nucleus to form ODVs and are occluded into OBs [2], which finally are released to the environment to initiate the next infection. The two phenotypes are genotypically identical, but have characteristic structural components which contribute to their respective functions [4, 5].

Mamestra brassicae NPV (MabrNPV) has a wide host range of over 30 species mainly in the family *Noctuidae* [6], and has been developed as a commercial biological insecticide in China. Recently, several baculoviral strains isolated from *Mamestra brassicae* [7], *Mamestra configurata* [8] and *Helicoverpa armigera* [9] have been sequenced and considered as MabrNPV variants. A MabrNPV variant (MabrNPV-CTa used in this study), was originally isolated from a naturally infected *Mamestra brassicae* in the Taian city, China in the 1970s, and classified into Group II lepidopteran NPV based on phylogenetic analysis of *polh*, *lef-8 and lef-9* [10]. The genome of MabrNPV-CTa was recently-sequenced and it consists of 153,890 bp encoding 165 predicted open reading frames (ORFs) (GenBank, KJ871680).

Mass spectrometry based proteomics strategy has been used widely to map protein components of virions. So far, several virion proteomics analysis of baculoviral BVs and ODVs have been performed, including Anticarsia gemmatalis multiple NPV (AgMNPV) BV and ODV [11], Autographa californica MNPV (AcMNPV) BV [12] and ODV [13], Bombyx mori NPV (BmNPV) ODV [14], Chrysodeixis chalcites NPV (ChchNPV) ODV [15], Helicoverpa armigera NPV (HearNPV) BV [16] and ODV [16, 17], Pieris rapae GV (PrGV) ODV [18] and Culex nigripalpus NPV (CuniNPV) ODV [19], and these studies facilitated our understanding on the structure and function of baculovirus virion. To obtain a comprehensive view of protein components of virion, our previous study applied multiple proteomic methodologies to analyze both BV and ODV of HearNPV [16].

The wide host range character of MabrNPV indicates that MabrNPV virions can be produced by different hosts. In this study, to obtain a more comprehensive view of MabrNPV virion proteome, we performed proteomic analysis on four types of MabrNPV-CTa virions: BVs and ODVs derived from infected *H*. *armigera*, and BVs and ODVs from *S*. *exigua*. A mass spectrometry with high scan speed was used, and, as a result, a total of 82 and 39 viral proteins were attributed to ODV and BV, respectively. Multiple host proteins were found to be associated with ODV and BV, and novel host proteins including two C-type lectin proteins were identified in BV. Furthermore, the identification of virion-associated host proteins should help us to better understand the mechanism of baculovirus infection.

## Methods and Materials

### Insects, virus infection and virion purification

Laboratory stocks of *H*. *armigera* and *S*. *exigua* larvae were reared [20] and the MabrNPV-CTa (IVCAS 1.50) from Wuhan Institute of Virology (Wuhan, People’s Republic of China) was propagated in both larvae. The larvae were infected at 3^rd^ instar by inoculation of 1×10^6^ OBs per larvae and the hemolymph were collected from infected larvae at 72 hours after infection and were diluted with 9 volume of 0.1×TE (TE, 10mM Tris, 1.0Mm EDTA, pH7.4), centrifuged at 5000g for 10 min at 4°C. BVs were isolated from hemolymph by the method used in purification of BVs propagated in cell culture [12]. OB purification and subsequent ODV isolation have been described previously [4].

### In-solution digestion of virion proteins

Proteins of purified BVs and ODVs derived from both host larval species, were precipitated with 3 volumes of 50% methanol/50% acetone/0.1% acetic acid. The pellets were resuspended in lysis buffer (4 mM CaCl_2_, 8 M urea, 0.2 M Tris-HCl [pH 8.0]), reduced with 10 mM DTT at 50°C for 30 min and alkylated with 40 mM iodoacetamide at room temperature in the dark for 30 min. The protein concentration was measured using the Bradford assay, and the proteins were digested with trypsin (Promega) at a ratio of 1:50 (trypsin/protein w/w). After incubated at 37°C overnight, the digested peptides were desalted using a SepPak C18 cartridge (Waters) and dried using a Speed Vac [21]. Two independent biological replicates were performed.

### Mass spectrometry analysis

All nano-ESI-based LC-MS/MS experiments were performed on a TripleTOF 5600+ System (AB SCIEX, USA) coupled with a splitless Ultra 1D Plus system (Eksigent, CA). The desalted peptides derived from 2 μg MabrNPV-CTa virions (BV or ODV) were dissolved in 0.1% formic acid/2% acetonitrile/98% H_2_O, loaded into a C18 trap column (5 μm, 5 x 0.3 mm, Agilent Technologies, Inc.) at a flow rate of 5 μL/min. The peptides were eluted to the C18 analytic column (75 μm × 150 mm, 3 μm particle size, 100 Å pore size, Eksigent) at a flow rate of 300 nL/min. A 100 min gradient was employed to separate peptides, and the mobile phase consisted of two components: component A was 3% DMSO/97% H_2_O with 0.1% formic acid, and component B was 3% DMSO/97% acetonitrile with 0.1% formic acid. IDA (information dependent acquisition) mode was used to acquire MS/MS. Survey scans were acquired in 250 ms and 40 product ion scans were collected in 50 ms/per scan. The precursor ion range was set from m/z 350 to m/z 1500, and the product ion range was set from m/z 100 to m/z 1500.

### Analysis of MS data for protein identification

Analysis of the raw MS spectra generated by LC-MS/MS analysis were submitted to the ProteinPilot 5.0 software program (AB SCIEX, USA), which uses the Paragon algorithm to perform protein identification [22]. The data analysis parameters were as follows: Sample type: Identification; Cys Alkylation: Iodoacetamide; Digestion: Trypsin; Instrument: TripleTOF 5600+; Special Factors: Urea denaturation; ID Focus: Biological modifications, Amino acid substitution; Search Effort: Thorough ID; Detected Protein Threshold [Unused ProtScore (Conf)]: 1.3 (95.0%). For the identification of viral and host proteins, an integrated database was used, which contains all the predicted ORFs of MabrNPV-CTa, lepidopteran protein database (derived from Genbank on Sep 30th, 2015).

## Results

### Viral proteins associated with ODV and BV

In this study, the MabrNPV-CTa BV and ODV virions propagated in both *H*. *armigera* and *S*. *exigua* larvae were used for proteomics analysis. Proteins extracted from purified virions were subjected to in-solution digestion, analyzed by LC-MS/MS and identified by ProteinPilot 5.0. The false discovery rates (FDRs) of peptide spectra matches determined by a decoy database search were <1%. Only proteins with at least one high confidence peptide (confidence score>99%) were considered as being identified. Two independent biological replicates were performed, and proteins identified in both biological replicates were used for further analysis.

**Fig 1:**
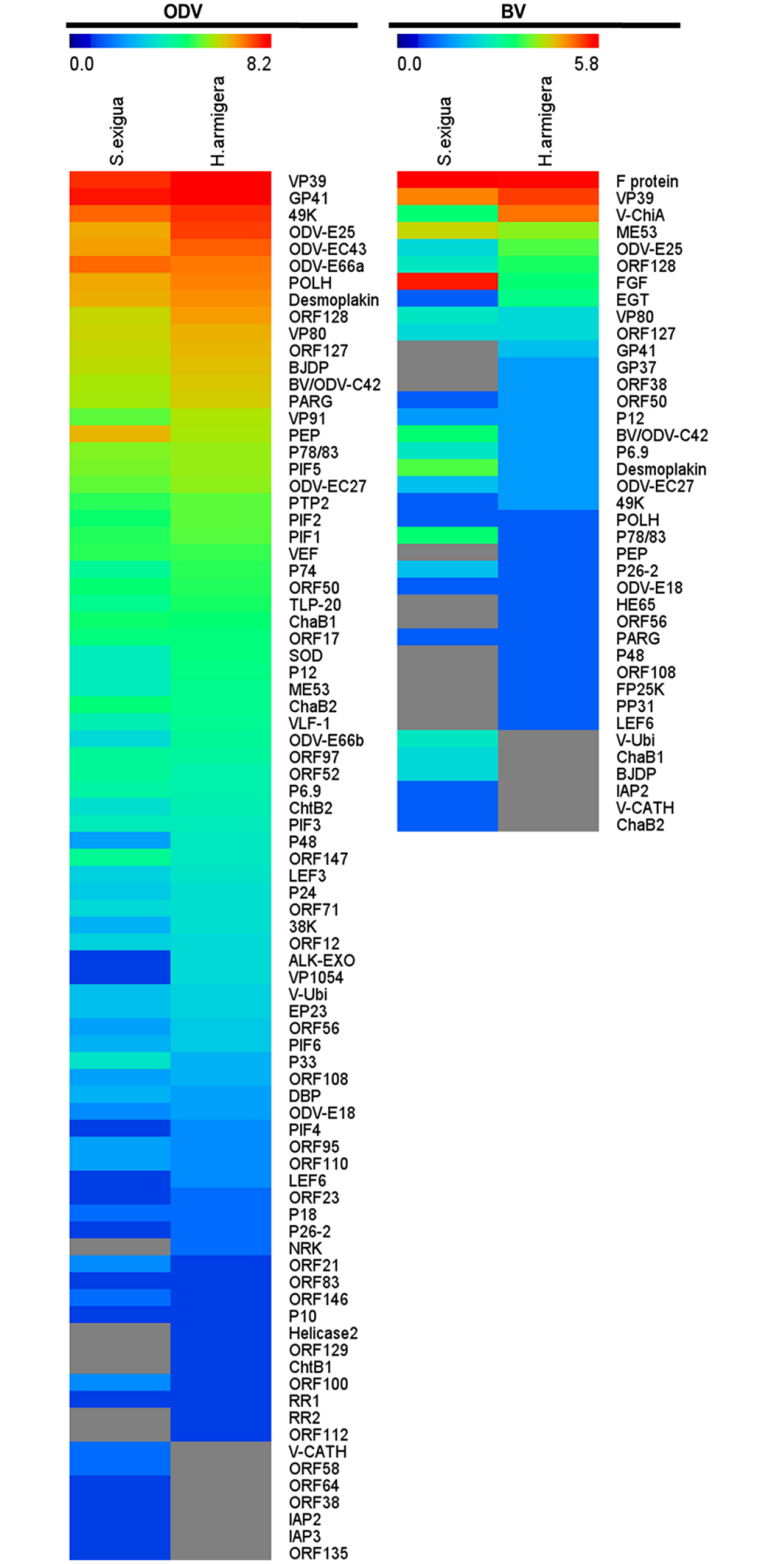
Overview of viral proteins associated with MabrNPV-CTa ODVs and BVs from different hosts. (Left) Proteins identified in ODVs produced from *H*. *armigera* and *S*. *exigua larvae*, (Right) Proteins identified in BVs produced from *H*. *armigera* and *S*. *exigua larvae*. Heat map was generated from log_2_(the number of matching peptides with confidence more than 99%) values reflecting identification of viral proteins from virions derived from different hosts. For the color scale limits in rainbow color maps, lower limit was set as 0 (minimum log_2_ value = 0), and upper limit was set as 8.2 (maximum log_2_ value = 8.2) for ODV while 5.8 (maximum log_2_ value = 5.8) for BV. The proteins not identified are indicated in gray.

The viral proteins identified in BVs or ODVs from different larvae were compared and demonstrated in Fig 1. In summary, a total of 82 viral proteins were identified as being associated with MabrNPV-CTa ODV (Fig 1A and S1 Table). Among these 82 proteins, 69 proteins were in ODVs produced in either larva. Six proteins including Helicase2, RR2, NRK, Mabr112, Mabr129 (AC53) and ChtB1 were only detected in ODVs from *H*. *armigera* larvae, and another 7 [V-CATH, Mabr38, Mabr58, Mabr64, IAP2, IAP3 and Mabr135 (AC43)] were only found in ODVs from *S*. *exigua* larvae (Fig 1A and S1 Table). Among a total of 39 viral proteins found in MabrNPV-CTa BV (Fig 1 and S2 Table), 22 were in BVs produced in either larva. Proteins found only in BVs from *H*. *armigera* were GP41, HE65, GP37, Mabr38 (AC4), PEP, Mabr56, P48, Mabr108 (AC75), FP25K, PP31 and LEF6 (Fig 1 and S2 Table), while 6 proteins including V-CATH, IAP2, ChaB2, ChaB1, BJDP and V-Ubi, were only found in BVs from *S*. *exigua* (Fig 1B and S2 Table). The identification of host specific proteins in both BVs and ODVs indicated that the protein components of virions might be affected by hosts. More host specific viral proteins were identified in BV virions, suggesting that the protein components of BV virion may be more host-dependent.

### Comparison of MabrNPV-CTa BV and ODV viral proteome

**Fig 2:**
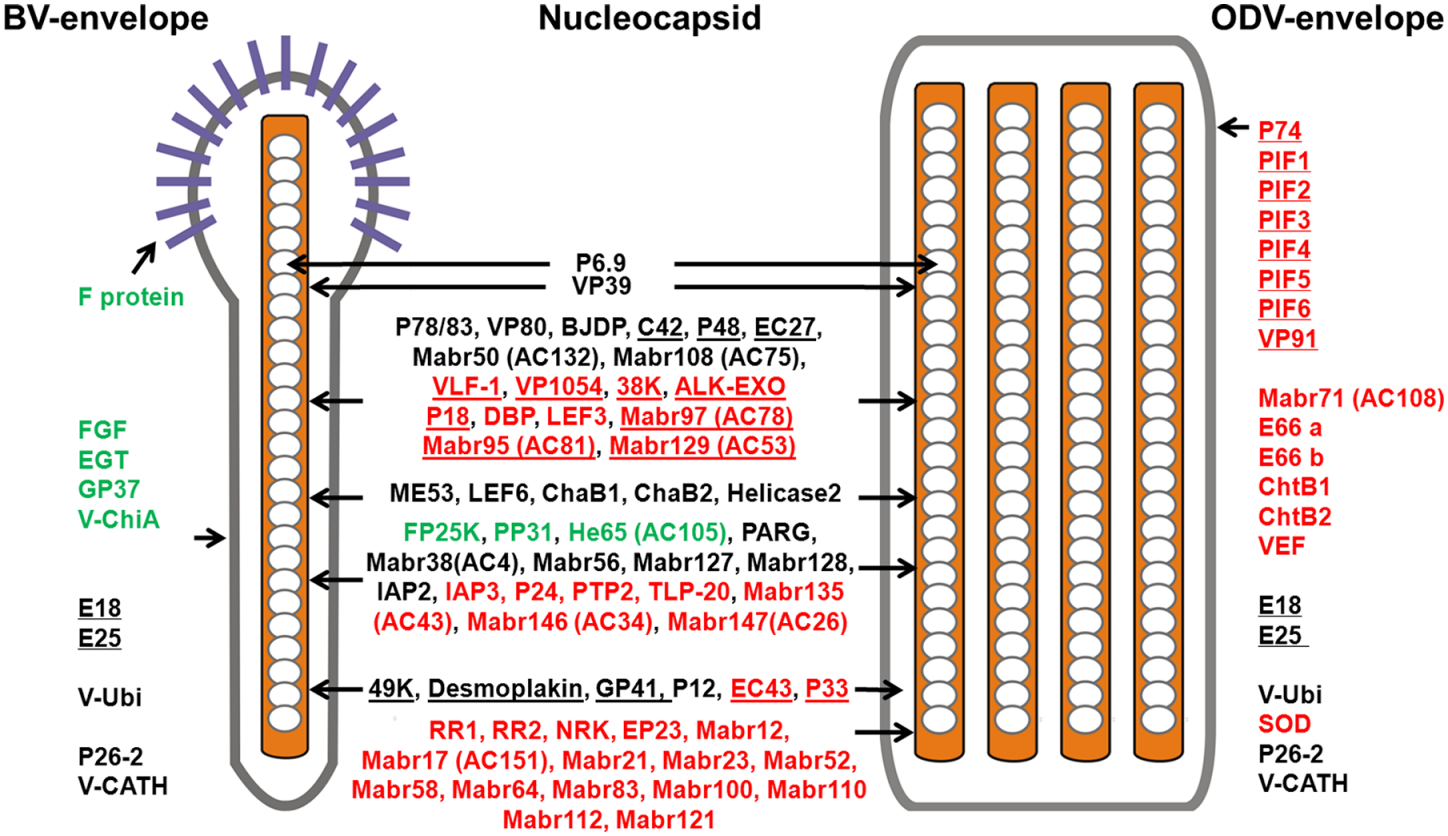
Overview of protein composition of MabrNPV-CTa BV and ODV. The proteins conserved in all sequenced baculoviruses are underlined. The proteins only identified in MabrNPV-CTa BVs or ODVs are indicated in green or red respectively. The ORF numbers of the AcMNPV homologues for the MabrNPV—CTa ORFs are indicated in brackets. Proteins including POLH, PEP and P10 were not listed here.

Among the identified virion-associated proteins, 50 were present only in ODV while 7 proteins were only in BV (Fig 1). Thirty-two proteins (including POLH and P10, not listed in Fig 2) were common to both BVs and ODVs (Fig 2), and some of these shared proteins were essential for nucleocapsid assembly and/or egress from nucleus (Fig 2 and S3 Table). For example, P6.9 is the nucleocapsid core protein binding DNA [23] while VP39 is the protein of nucleocapsid sheet [24], and both are the major nucleocapsid proteins. BJDP has been found as both BV and ODV associated proteins [25] and was indispensable for virion production [26]. P78/83 is associated with assembly and cellular trafficking of nucleocapsid [27, 28]. P12 is essential for mediating nuclear localization of G-Actin and BV production [29]. Proteins including 49K, GP41, Desmoplakin (AC66) have been reported as ODV tegument proteins and nucleocapsid associated proteins of BV, and are essential for nucleocapsid egress from nucleus to cytoplasm [[30], S3 Table]. Additionally, Mabr50 (AC132) [26], Mabr108 (AC75) [31] and ODV-E18 [32] have been demonstrated to be important for the production of infectious BVs.

Viral proteins with other function were also found in both BV and ODV (Fig 2 and S3 Table), such as *i*) proteins that can affect production (ODV-E25, P26-2) [33–35] and release (V-CATH) of mature polyhedra [36] (Figs 1 and 2), *ii*) non-essential proteins that are involved in DNA replication (ME53) [37], late protein expression (LEF6) [38] and DNA binding (ChaB1, ChaB2) [26, 39–41] (Fig 2 and S3 Table), *iii*) other non-essential proteins with uncharacterized functions but have been reported as virion-associated [5, 12, 15], including V-Ubi, PARG, Mabr127 (HA45), Mabr128 (HA44) and IAP2 (Fig 2; the functional annotation of these proteins was attached in S3 Table).

ODV and BV use specific envelope proteins to entry into host cells. P74, PIF1-6 and VP91 are essential for ODV oral infection (Fig 2 and S3 Table), and all are present in MabrNPV-CTa- propagated ODVs (Figs 1 and 2). ODV-66 has chondroitinase activity and was proposed to destroy the peritrophic matrix of the host’s midgut, thus facilitating ODV infection [42]. The ChtBs (AC145 homologues) and VEF have functional roles in the degradation of the host’s peritrophic matrix to enhance ODV virulence. These three proteins were only found in ODVs in this study. F protein, required for BV entry into host cells and cell-cell transmission of infection, was identified as a BV specific protein.

One protein that drew our attention was the Mabr71 (AC108), which was only identified in ODV from both two types of larvae. This protein has a transmembrane domain (32-53aa) and its homologue, AC108, has been found in PIF complex [43], Furthermore, other two homologues of Mabr71, SF58 and Bm91, were demonstrated to be involved in the ODV infection processes [44, 45].

### Comparison of MabrNPV-CTa virion proteome with other baculovirus proteomic studies

In this study, proteomic analysis of MabrNPV-CTa was performed and we identified a total of 82 ODV proteins and 39 BV proteins respectively (Fig 2). In comparison with previous virion proteomic analyses of 7 baculoviruses (proteome of 7 ODVs and 3 BVs; S3 Table), it seemed that most of the core proteins are shared by ODVs, while shared BV and ODV conserved proteins appear to be mainly involved in assembly and egress of nucleocapsid, DNA replication and RNA transcription (S3 Table). For example, the VP80 is essential for egress of nucleocapsid from the nucleus [46] and the P78/83 is required for nucleocapsid transport in cytoplasm [27, 28]. FP25K, a protein involved in regulation BV/ODV production [47–50], was also found in all the BVs and ODVs with the exception of CuniNPV ODV and MabrNPV ODV. Although proteins important for oral infection are mostly ODV-specific, PIF4 has been identified in AcMNPV BV [51].

In addition, by referring to previous proteomic studies, 23 proteins in this study were identified as novel virion-associated proteins, including RR1, RR2, NRK, EP23, TLP-20, HE65 (AC105), Mabr12, Mabr17 (AC151), Mabr21, Mabr23, Mabr38 (AC4), Mabr52, Mabr56, Mabr58, Mabr64, Mabr83, Mabr100, Mabr110 (AC151), Mabr112, Mabr127, Mabr128, and Mabr135 (AC43). Among these proteins, seven proteins were shared by both BV and ODV from MabrNPV-CTa, including Mabr12, Mabr17 (AC151), Mabr21, Mabr38 (AC4), Mabr56, Mabr127 (HA45) and Mabr128 (HA44) (Fig 2). The identification of these proteins could improve the annotation of baculovirus genome.

### Virion-associated host proteins

**Fig 3:**
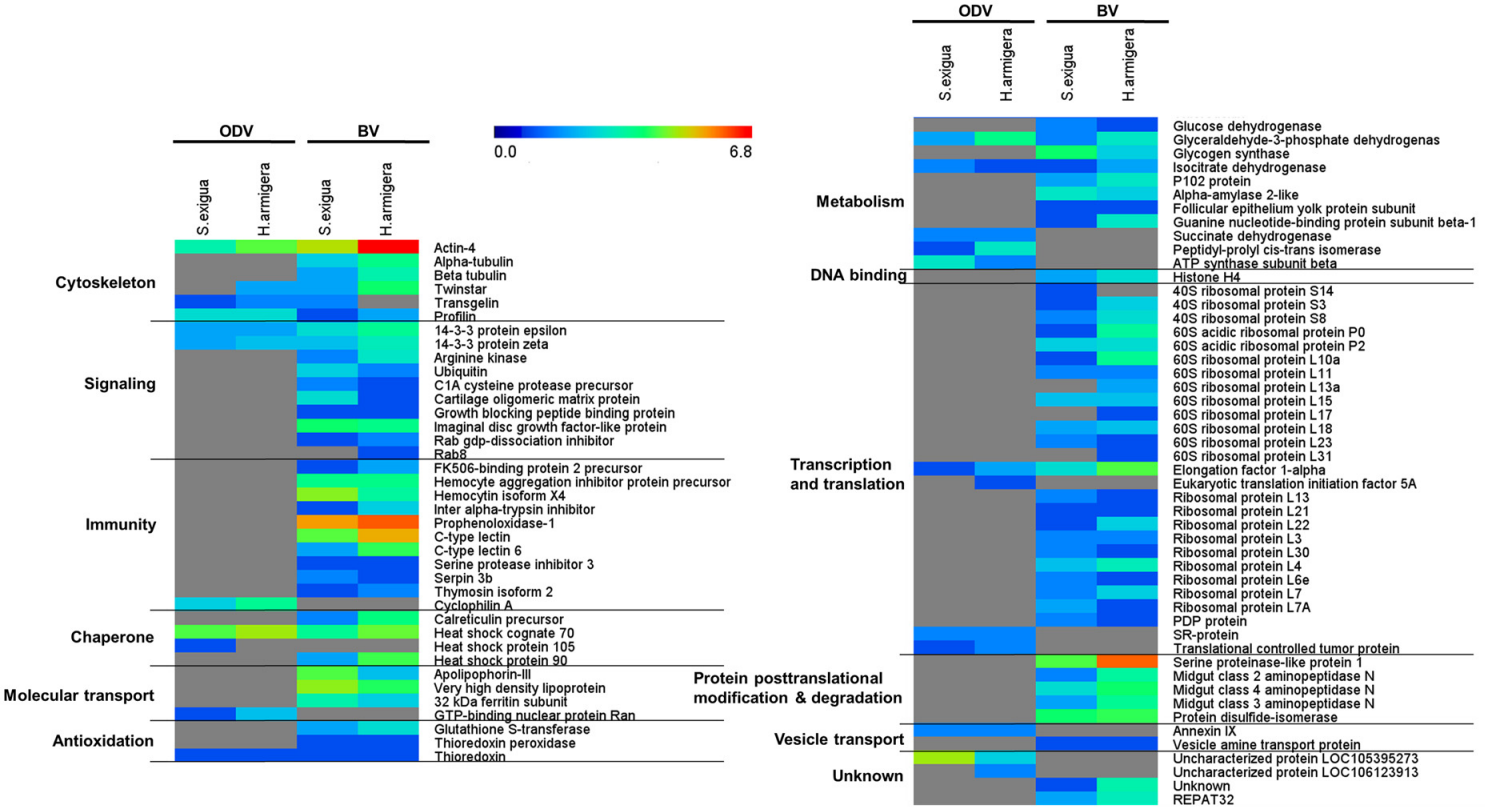
Overview of host proteins associated with MabrNPV-CTa ODVs and BVs from different hosts. Heat map was generated from log_2_(the number of matching peptides with confidence more than 99%) values reflecting identification of host proteins from virions derived from different hosts. For the color scale limits in rainbow color maps, lower limit was set as 0 (minimum log_2_ value = 0), and upper limit was set as 6.8 (maximum log_2_ value = 6.8). The proteins not identified are indicated in gray.

During replication, host proteins can be incorporated in virion, some of which may have roles in the infection process [52]. By searching mass spectra against the lepidopteran protein database, 88 host proteins were identified to be virion-associated. Among these, 23 host proteins were identified as ODV-associated (Fig 3 and S4 Table). Based on their involvement in cellular structure and functions, these host proteins can be classified into different categories including i) cytoskeleton, such as Actin, Profilin, Transgelin and Twinstar, ii) signaling, containing 14-3-3 protein epsilon and 14-3-3 protein zeta, iii) immunity, including Cyclophilin A, iv) chaperone (Heat shock cognate 70 protein and Heat shock protein 105), v) molecular transport (GTP-binding nuclear protein Ran), vi) antioxidation (Thioredoxin), vii) metabolism (5 proteins) and viii) transcription and translation (4 proteins) and two proteins with uncharacterized function(s).

Seventy-six host proteins were found in both BVs from the two different hosts. Host proteins identified in BVs have their involvement in cellular structure and functions, including *i*) cytoskeleton, *ii*) signaling, *iii*) immunity, *iv*) chaperone, *v*) molecular transport, *vi*) antioxidation, *vii*) metabolism, *viii*) DNA binding, *ix*) transcription and translation, *x*) post-translational modification, *xi*) vesicle transport and *xii*) posttranslational modification and degradation and two proteins with uncharacterized function(s) (Fig 3, for detailed information, see S5 Table). Little overlaps of hosts proteins were found between BVs and ODVs, however, host proteins identified in BVs and ODVs were enriched in similar cellular structure and function categories (Fig 3), suggesting that these cellular structures and functions play roles in both two distinct life cycles of MabrNPV-CTa.

## Discussion

In this study, using a TripleTOF 5600+ System, a proteomic analysis of different virions of MabrNPV-CTa, a Group II *lepidopteran* NPV was conducted. Considering that MabrNPV has a wide host range, we performed proteomic analysis on MabrNPV BVs and ODVs propagated in *H*. *armigera* and *S*. *exigua* larvae, aiming to find more virion-associated proteins and thus obtain a more comprehensive view of MabrNPV virion proteome. As a result, a total of 82 ODV- and 39 BV- proteins were identified, and the number of proteins identified here was comparable to our previous comprehensive study on HearNPV virions, where multiple proteomic methodologies were employed [5]. Among all 162 hypothetical proteins in MabrNPV genome, 92 proteins were identified in progeny virons here, and 23 proteins were firstly identified as virion-associated. These novel virions associated proteins showed low similarity with their homologues in other baculoviruses [9]. The identification of these proteins confirmed their expression during viral replication cycle, and can facilitate the annotation of the genome of MabrNPV-CTa and other baculoviruses.

The majority of baculovirus virion proteomics studies were performed on ODVs and with improved technologies, the numbers of ODV-associated proteins have been on the increase [5, 12–15, 17–19] (S3 Table). In this proteomic analysis of MabrNPV-CTa ODV, a total of 82 associated proteins were identified, 69 of which were common to ODVs from *H*. *armigera* and *S*. *exigua* (Fig 1 and S2 Table). Six viral proteins (Helicase2, RR2, NRK, Mabr112, Mabr129 (AC53) and ChtB1) were only identified in ODV virions from *H*. *armigera* and 7 proteins [V-CATH, Mabr38, Mabr58, Mabr64, IAP2, IAP3 and Mabr135 (AC43)] were only identified in that from *S*. *exigua*. LC-MS/MS analysis identified a total of 39 viral proteins in BV, 22 of which were common to BVs produced in either *H*. *armigera* or *S*. *exigua*. Most of the common proteins are essential for nucleocapsid assembly and transport (Fig 1A and S3 Table). Seventeen viral proteins were identified in BV from either *H*. *armigera* or *S*. *exigua*. Most of these, with the exception of GP41 and BJDP, are not essential for viral replication (S3 Table). It should be noted that BV demonstrated remarkable flexibility for efficient incorporation of non-specific proteins when used as baculovirus surface display systems [53]. Many hitherto unknown factors may contribute to the variation in BV-associated proteins. The identification of host specific proteins suggested that protein components of virions derived from different hosts are not identical. However, how protein components of virions were affected by different hosts is still not fully understood, and further studies are needed to explain this phenomenon.

### The association of host proteins with baculovirus virions suggests their involvement in viral infection

Besides the viral encoded proteins, 88 host proteins were also identified in BVs or ODVs. A total of 23 host proteins associated with ODV but, in contrast, 76 host proteins were associated with BV. Less host proteins were found in ODVs and most of these host proteins were identified with no more than three peptides by LC-MS/MS analysis of ODVs from either host (S4 Table). The phenomenon that there are less host proteins associated with ODV than with BV was also observed in our previous proteomic analysis study [5]. We found the majority of host proteins were identified in both *H*. *armigera* and *S*. *exigua* derived virions, suggesting that the differences between host-specific host proteins might be not significant (Fig 3). The identification of these host proteins in common confirmed their existence in virions, and further studies on these proteins may help us to better understand the baculovirus-host interactions.

Among the host proteins identified in BVs and ODVs, actin can be recruited for the cellular transport of nucleocapsids during infection [28]. Some actin dynamics regulation proteins, such as GTPases, ADF and 14-3-3 ζ, were also found in both BV and ODV. It has been reported that 14-3-3 ζ can regulates actin dynamics by stabilizing phosphorylated cofilin [54]. Cyclophilin A and heat shock protein 70, identified here, have been reported to be specifically incorporated into lentivirus and function in different steps of virus infection.

Multiple host proteins, such as cytoskeleton proteins, signaling proteins, vesicle transport proteins and signaling proteins, have been found in MabrNPV-CTa BV and HearNPV BV produced *in vivo* and *in vitro* respectively, as in the case of other envelope viruses [5]. This would imply that enveloped viruses might use certain common or similar cellular pathways during their infection processes. Furthermore, 10 host proteins involved in immune response processes were identified in BV, including two lectin proteins which were only found in MabrNPV BV. It has been reported that one member of C-type lectin family can be induced by West Nile Virus (WNV) and collaborates with a CD45 phosphatase to facilitate WNV infection in midgut and other tissues of mosquitoes. And several C-type lectins appear to facilitate entry of multiple viruses, such as HCV [55] Ebola virus, Hendra virus, Nipah virus [56] and Marburg Virus [57]. The C-type lectin was firstly identified in BV produced in vivo. It would be interesting to investigate the function of this protein in baculovirus infection.

Some BV associated host proteins that participate in cellular processes, such as transcription and translation, antioxidation, metabolism (S5 Table), might be involved in baculoviral infection, suggesting an intricate evolutionary relationship between the virus and the host and that the parasite did not need to develop a coding capacity for proteins already available in permissive hosts. We also found aminopeptidases in BV. Aminopeptidases are mostly found in midgut microvillar membrane[58], but their presence in hemocoelic tissue[59, 60] and in fat body[61] has also been reported.

Taken together, our study outlined a comprehensive view of viral and host proteins associated with MabrNPV BVs and ODVs produced in two different host species. This should help us to understand and have a second view of virion structure and its interactions with host proteins, and should promote investigations on baculovirus infection mechanisms in relationship to host proteins.

## Supporting Information

S1 Table Identification of viral proteins associated with MabrNPV-CTa ODV. (DOCX)

S2 Table Identification of viral proteins associated with MabrNPV-CTa BV. (DOCX)

S3 Table Summary of proteomic studies of baculoviral BVs and ODVs. (DOCX)

S4 Table Identification of host proteins associated with MabrNPV-CTa ODV. (DOCX)

S5 Table Identification of host proteins associated with MabrNPV-CTa BV. (DOCX)
