# Supplementary material for: Proteomic Analysis of Mamestra Brassicae Nucleopolyhedrovirus Progeny Virions from Two Different Hosts
Source: PLoS One. 2016 Apr 8;11(4):e0153365. doi: 10.1371/journal.pone.0153365 (PMC4825930; doi:10.1371/journal.pone.0153365)
Supplement: S1 Table — (DOCX) [file pone.0153365.s001.docx]

**S1 Table. Identification of viral proteins associated with MabrNPV-CTa ODV.**

| **No.** | **Accession #** | **Protein** |  | **Viral ORF** | |  |  | **ODV from *H.armigera*** | | |  |  | **ODV from *S.exigua*** | | |  |
| --- | --- | --- | --- | --- | --- | --- | --- | --- | --- | --- | --- | --- | --- | --- | --- | --- |
|  |  |  | **MabrNPV-CTa** | | **AcMNPV** | | **Score^a^** | | **% Cov^b^** | **Peptides^c^** | | **Score^a^** | | **% Cov^b^** | **Peptides^c^** | |
| **1** | **gi\|674653849** | **POLH** | **1** | | **8** | | **52.01** | | **82.1** | **131** | | **57.36** | | **84.2** | **99** | |
| **2** | **gi\|674653850** | **P78/83** | **2** | | **9** | | **39.67** | | **48.5** | **54** | | **47.06** | | **48.7** | **48** | |
| **3** | **gi\|674653854** | **PIF5** | **6** | | **148** | | **19.56** | | **46.4** | **54** | | **31.48** | | **45** | **46** | |
| **4** | **gi\|674653855** | **ME53** | **7** | | **139** | | **26.36** | | **43.8** | **21** | | **25.79** | | **37** | **14** | |
| **5** | **gi\|674653860** | **P24** | **11** | | **129** | | **9.17** | | **33.8** | **11** | | **8** | | **28.4** | **8** | |
| **6** | **gi\|674653861** | **Mabr12** | **12** | | **-** | | **6.83** | | **49.5** | **10** | | **6.43** | | **61.2** | **9** | |
| **7** | **gi\|674653866** | **Mabr17** | **17** | | **151** | | **20.38** | | **61** | **24** | | **19.67** | | **61** | **24** | |
| **8** | **gi\|674653870** | **Mabr21** | **21** | | **-** | | **2.58** | | **8.9** | **2** | | **4.46** | | **16.7** | **4** | |
| **9** | **gi\|674653872** | **Mabr23** | **23** | | **-** | | **4.05** | | **49** | **3** | | **3.16** | | **42.3** | **2** | |
| **10** | **gi\|674653874** | **Helicase2** | **25** | | **-** | | **3.55** | | **7.9** | **2** | | **-** | | **-** | **-** | |
| **11** | **gi\|674653876** | **V-CATH** | **27** | | **127** | | **-** | | **-** | **-** | | **2.73** | | **8.2** | **3** | |
| **12** | **gi\|674653881** | **PTP2** | **32** | | **1** | | **25.04** | | **65.9** | **40** | | **25.37** | | **65.9** | **32** | |
| **13** | **gi\|674653886** | **ChtB2** | **37** | | **145** | | **10.55** | | **65.8** | **15** | | **10.15** | | **62.3** | **11** | |
| **14** | **gi\|674653887** | **Mabr38** | **38** | | **4** | | **-** | | **-** | **-** | | **2.08** | | **6.9** | **1** | |
| **15** | **gi\|674653891** | **PIF2** | **42** | | **22** | | **32.03** | | **60.6** | **40** | | **32.99** | | **51.6** | **28** | |
| **16** | **gi\|674653892** | **PIF1** | **43** | | **119** | | **37.14** | | **49.3** | **39** | | **30.02** | | **48.2** | **31** | |
| **17** | **gi\|674653896** | **ALK-EXO** | **47** | | **133** | | **11.18** | | **20.5** | **10** | | **4** | | **7.3** | **2** | |
| **18** | **gi\|674653899** | **Mabr50** | **50** | | **132** | | **23.59** | | **75.9** | **31** | | **20.57** | | **68.4** | **26** | |
| **19** | **gi\|674653900** | **RR2** | **51** | | **-** | | **2.03** | | **5.1** | **1** | | **-** | | **-** | **-** | |
| **20** | **gi\|674653901** | **Mabr52** | **52** | | **-** | | **17.57** | | **37.5** | **16** | | **17.6** | | **45.3** | **19** | |
| **21** | **gi\|674653902** | **PEP** | **53** | | **131** | | **23.68** | | **52.9** | **59** | | **45.98** | | **58.8** | **92** | |
| **22** | **gi\|674653905** | **Mabr56** | **56** | | **-** | | **5.16** | | **30.4** | **8** | | **6.98** | | **25** | **5** | |
| **23** | **gi\|674653906** | **SOD** | **57** | | **31** | | **9.3** | | **88.1** | **24** | | **11.47** | | **86.8** | **14** | |
| **24** | **gi\|674653907** | **Mabr58** | **58** | | **-** | | **-** | | **-** | **-** | | **2.85** | | **33.1** | **3** | |
| **25** | **gi\|674653908** | **PIF3** | **59** | | **115** | | **15.38** | | **64** | **14** | | **17.19** | | **69** | **14** | |
| **26** | **gi\|674653910** | **PARG** | **61** | | **-** | | **69.23** | | **80.2** | **77** | | **66.72** | | **73.8** | **57** | |
| **27** | **gi\|674653912** | **NRK** | **63** | | **33** | | **4.24** | | **8.7** | **3** | | **-** | | **-** | **-** | |
| **28** | **gi\|674653913** | **Mabr64** | **64** | | **4** | | **-** | | **-** | **-** | | **2.44** | | **15.8** | **2** | |
| **29** | **gi\|674653919** | **ODV-E66a** | **70** | | **46** | | **55.32** | | **70.7** | **135** | | **89.81** | | **74.7** | **149** | |
| **30** | **gi\|674653920** | **Mabr71** | **71** | | **108** | | **7.34** | | **44.7** | **11** | | **7.17** | | **44.7** | **10** | |
| **31** | **gi\|674653921** | **ODV-EC43** | **72** | | **109** | | **69.44** | | **89.9** | **159** | | **69.48** | | **82.6** | **106** | |
| **32** | **gi\|674653923** | **VP80** | **74** | | **104** | | **71.87** | | **67.7** | **96** | | **70.08** | | **64.1** | **72** | |
| **33** | **gi\|674653924** | **P48** | **75** | | **103** | | **18.59** | | **28.4** | **13** | | **8.98** | | **15.4** | **5** | |
| **34** | **gi\|674653925** | **P12** | **76** | | **102** | | **12.84** | | **70.6** | **23** | | **11.13** | | **65.7** | **14** | |
| **35** | **gi\|674653926** | **BV/ODV-C42** | **77** | | **101** | | **39.98** | | **70.6** | **80** | | **42.5** | | **61** | **59** | |
| **36** | **gi\|674653927** | **P6.9** | **78** | | **100** | | **3.13** | | **14.3** | **16** | | **4.18** | | **14.3** | **17** | |
| **37** | **gi\|674653929** | **38K** | **80** | | **98** | | **11.77** | | **35** | **11** | | **8.21** | | **20** | **6** | |
| **38** | **gi\|674653930** | **VEF** | **81** | | **-** | | **36.75** | | **40.1** | **34** | | **45.26** | | **40.8** | **32** | |
| **39** | **gi\|674653932** | **Mabr83** | **83** | | **-** | | **2.08** | | **24.7** | **2** | | **2.01** | | **11.3** | **1** | |
| **40** | **gi\|674653933** | **PIF4** | **84** | | **96** | | **6.03** | | **38.4** | **4** | | **2.76** | | **34.3** | **2** | |
| **41** | **gi\|674653935** | **ODV-E25** | **86** | | **94** | | **53.3** | | **65.7** | **189** | | **47.29** | | **65.7** | **99** | |
| **42** | **gi\|674653936** | **P18** | **87** | | **93** | | **2.12** | | **8.1** | **3** | | **2** | | **8.1** | **3** | |
| **43** | **gi\|674653937** | **P33** | **88** | | **92** | | **7.8** | | **11.9** | **6** | | **16.2** | | **34.9** | **12** | |
| **44** | **gi\|674653940** | **VP39** | **91** | | **89** | | **80.56** | | **98.5** | **285** | | **85.36** | | **96.4** | **209** | |
| **45** | **gi\|674653942** | **VP91** | **93** | | **83** | | **52.38** | | **49.5** | **60** | | **45.67** | | **39** | **40** | |
| **46** | **gi\|674653943** | **TLP-20** | **94** | | **82** | | **17.35** | | **56.9** | **29** | | **18.05** | | **62.6** | **21** | |
| **47** | **gi\|674653944** | **Mabr95** | **95** | | **81** | | **6.03** | | **18.8** | **4** | | **10.64** | | **28.8** | **5** | |
| **48** | **gi\|674653945** | **GP41** | **96** | | **80** | | **68.37** | | **94.6** | **279** | | **81.26** | | **92.2** | **240** | |
| **49** | **gi\|674653946** | **Mabr97** | **97** | | **78** | | **9.36** | | **33.1** | **18** | | **6.23** | | **24.5** | **19** | |
| **50** | **gi\|674653947** | **VLF-1** | **98** | | **77** | | **17.2** | | **29.7** | **20** | | **20.17** | | **28.2** | **15** | |
| **51** | **gi\|674653949** | **Mabr100** | **100** | | **-** | | **2** | | **3.6** | **1** | | **4.41** | | **8.2** | **4** | |
| **52** | **gi\|674653951** | **IAP2** | **102** | | **71** | | **-** | | **-** | **-** | | **2.02** | | **6** | **1** | |
| **53** | **gi\|674653953** | **PIF6** | **104** | | **68** | | **8** | | **28.9** | **8** | | **6** | | **24.8** | **6** | |
| **54** | **gi\|674653954** | **LEF3** | **105** | | **67** | | **11.55** | | **25.5** | **12** | | **10.94** | | **19.6** | **9** | |
| **55** | **gi\|674653955** | **Desmoplakin** | **106** | | **66** | | **63.65** | | **39.6** | **119** | | **88.36** | | **48.3** | **97** | |
| **56** | **gi\|674653957** | **Mabr108** | **108** | | **75** | | **4.16** | | **31** | **6** | | **6.06** | | **42.6** | **5** | |
| **57** | **gi\|674653959** | **Mabr110** | **110** | | **151** | | **3.56** | | **37** | **4** | | **7.77** | | **31.7** | **5** | |
| **58** | **gi\|674653961** | **Mabr112** | **112** | | **-** | | **2.06** | | **5.2** | **1** | | **-** | | **-** | **-** | |
| **59** | **gi\|674653970** | **ChaB2** | **120** | | **60** | | **12.12** | | **45.1** | **21** | | **14.05** | | **63.7** | **25** | |
| **60** | **gi\|674653971** | **ChaB1** | **121** | | **58-59** | | **12.96** | | **44.4** | **26** | | **18.2** | | **50.3** | **28** | |
| **61** | **gi\|674653975** | **VP1054** | **125** | | **54** | | **5.17** | | **27.7** | **10** | | **4.33** | | **16.4** | **2** | |
| **62** | **gi\|674653977** | **Mabr127** | **127** | | **-** | | **21.9** | | **88** | **91** | | **24.95** | | **78.7** | **69** | |
| **63** | **gi\|674653978** | **Mabr128** | **128** | | **-** | | **41.86** | | **58.5** | **110** | | **44.06** | | **59.2** | **70** | |
| **64** | **gi\|674653979** | **Mabr129** | **129** | | **53** | | **2.28** | | **21.7** | **2** | | **-** | | **-** | **-** | |
| **65** | **gi\|674653981** | **IAP3** | **131** | | **27** | | **-** | | **-** | **-** | | **2.15** | | **3.2** | **1** | |
| **66** | **gi\|674653982** | **BJDP** | **132** | | **51** | | **47.4** | | **54.7** | **85** | | **48.3** | | **55** | **65** | |
| **67** | **gi\|674653985** | **Mabr135** | **135** | | **43** | | **-** | | **-** | **-** | | **2.06** | | **24.2** | **1** | |
| **68** | **gi\|674653986** | **ODV-E66b** | **136** | | **46** | | **24.47** | | **21.6** | **19** | | **14.2** | | **14.3** | **10** | |
| **69** | **gi\|674653995** | **V-Ubi** | **145** | | **35** | | **7.22** | | **40** | **9** | | **10.35** | | **55** | **7** | |
| **70** | **gi\|674653996** | **Mabr146** | **146** | | **34** | | **2.54** | | **16** | **2** | | **5.87** | | **21.6** | **3** | |
| **71** | **gi\|674653997** | **Mabr147** | **147** | | **26** | | **9.34** | | **71.2** | **13** | | **11.67** | | **81.4** | **20** | |
| **72** | **gi\|674653998** | **DBP** | **148** | | **25** | | **5.58** | | **18.4** | **5** | | **7.95** | | **20.6** | **6** | |
| **73** | **gi\|674653999** | **LEF6** | **149** | | **28** | | **5.07** | | **22.7** | **4** | | **3.55** | | **14.2** | **2** | |
| **74** | **gi\|674654001** | **P26-2** | **151** | | **136** | | **5.64** | | **17.3** | **3** | | **2.18** | | **4.5** | **1** | |
| **75** | **gi\|674654002** | **P10** | **152** | | **137** | | **4** | | **34.9** | **2** | | **2.01** | | **14.5** | **1** | |
| **76** | **gi\|674654003** | **P74** | **153** | | **138** | | **27.74** | | **29.5** | **32** | | **29.83** | | **28.9** | **19** | |
| **77** | **gi\|674654006** | **EP23** | **156** | | **146** | | **10** | | **30.9** | **9** | | **9.56** | | **31.9** | **7** | |
| **78** | **gi\|674654007** | **ChtB1** | **157** | | **145** | | **2** | | **14.1** | **2** | | **-** | | **-** | **-** | |
| **79** | **gi\|674654008** | **ODV-EC27** | **158** | | **143** | | **37.84** | | **78.1** | **51** | | **36.96** | | **75.9** | **41** | |
| **80** | **gi\|674654009** | **ODV-E18** | **159** | | **144** | | **2** | | **12.9** | **5** | | **4.24** | | **37.7** | **4** | |
| **81** | **gi\|674654010** | **49K** | **160** | | **142** | | **91.21** | | **80.7** | **203** | | **87.22** | | **83.7** | **152** | |
| **82** | **gi\|674654012** | **RR1** | **162** | | **-** | | **2** | | **1.8** | **1** | | **2.03** | | **1.7** | **1** | |

**^a^ Score was given by ProteinPilot software. The Score value is calculated by the following formula: Score = -log(1-PercentConfidence/100). Protein identitied with Score higher than 1.3 (p<0.05) were considered significant and listed in this table.**

**^b^ The percentage of matching amino acids of identified peptides with confidence greater than 95% divided by the total number of amino acids in the sequence.**

**^c^ The number of matching peptides with confidence more than 95%.**
