# Supplementary material for: Proteomic Analysis of Mamestra Brassicae Nucleopolyhedrovirus Progeny Virions from Two Different Hosts
Source: PLoS One. 2016 Apr 8;11(4):e0153365. doi: 10.1371/journal.pone.0153365 (PMC4825930; doi:10.1371/journal.pone.0153365)
Supplement: S2 Table — (DOCX) [file pone.0153365.s002.docx]

**S2 Table. Identification of viral proteins associated with MabrNPV-CTa BV.**

| **No.** | **Accession #** | **Protein** |  | **Viral ORF** | |  |  | **ODV from *H.armigera*** | | |  |  | **ODV from *S.exigua*** | | |  |
| --- | --- | --- | --- | --- | --- | --- | --- | --- | --- | --- | --- | --- | --- | --- | --- | --- |
|  |  |  | **MabrNPV-CTa** | | **AcMNPV** | | **Score^a^** | | **% Cov^b^** | **Peptides^c^** | | **Score^a^** | | **% Cov^b^** | **Peptides^c^** | |
| **1** | **gi\|674653849** | **POLH** | **1** | | **8** | | **2.11** | | **5.7** | **2** | | **4** | | **11** | **2** | |
| **2** | **gi\|674653850** | **P78/83** | **2** | | **9** | | **3.25** | | **4.8** | **2** | | **19.51** | | **22.4** | **10** | |
| **3** | **gi\|674653855** | **ME53** | **7** | | **139** | | **21.88** | | **33.6** | **16** | | **34.9** | | **51.4** | **20** | |
| **4** | **gi\|674653857** | **F protein** | **8** | | **23** | | **42.9** | | **30.1** | **50** | | **66.85** | | **43.2** | **57** | |
| **5** | **gi\|674653867** | **V-ChiA** | **18** | | **126** | | **36.47** | | **48.8** | **33** | | **17.31** | | **25.1** | **10** | |
| **6** | **gi\|674653875** | **HE65** | **26** | | **105** | | **2.45** | | **3.1** | **1** | | **-** | | **-** | **-** | |
| **7** | **gi\|674653876** | **V-CATH** | **27** | | **127** | | **-** | | **-** | **-** | | **2** | | **2.6** | **1** | |
| **8** | **gi\|674653880** | **GP37** | **31** | | **64** | | **5.85** | | **19.1** | **3** | | **-** | | **-** | **-** | |
| **9** | **gi\|674653882** | **EGT** | **33** | | **15** | | **10.58** | | **10.2** | **9** | | **4.13** | | **5.9** | **2** | |
| **10** | **gi\|674653887** | **Mabr38** | **38** | | **4.00** | | **4** | | **17.1** | **3** | | **-** | | **-** | **-** | |
| **11** | **gi\|674653894** | **FGF** | **45** | | **32** | | **8.86** | | **30** | **10** | | **36.21** | | **57.4** | **47** | |
| **12** | **gi\|674653899** | **Mabr50** | **50** | | **132** | | **2.15** | | **21.1** | **3** | | **4.23** | | **18.1** | **2** | |
| **13** | **gi\|674653902** | **PEP** | **53** | | **131** | | **2.44** | | **3.7** | **2** | | **-** | | **-** | **-** | |
| **14** | **gi\|674653905** | **Mabr56** | **56** | | **-** | | **2** | | **8.8** | **1** | | **-** | | **-** | **-** | |
| **15** | **gi\|674653910** | **PARG** | **61** | | **-** | | **2.12** | | **1.9** | **1** | | **4.18** | | **4.3** | **2** | |
| **16** | **gi\|674653923** | **VP80** | **74** | | **104** | | **8.92** | | **12.6** | **5** | | **12.02** | | **15.9** | **6** | |
| **17** | **gi\|674653924** | **P48** | **75** | | **103** | | **2.33** | | **4.5** | **1** | | **-** | | **-** | **-** | |
| **18** | **gi\|674653925** | **P12** | **76** | | **102** | | **2.49** | | **31.4** | **3** | | **6.01** | | **42.2** | **3** | |
| **19** | **gi\|674653926** | **BV/ODV-C42** | **77** | | **101** | | **6.18** | | **12.6** | **3** | | **19.54** | | **33.5** | **10** | |
| **20** | **gi\|674653927** | **P6.9** | **78** | | **100** | | **2.41** | | **13** | **3** | | **4** | | **13** | **6** | |
| **21** | **gi\|674653935** | **ODV-E25** | **86** | | **94** | | **10.86** | | **47.7** | **13** | | **9.01** | | **39.4** | **5** | |
| **22** | **gi\|674653940** | **VP39** | **91** | | **89** | | **28.27** | | **63.5** | **41** | | **27.98** | | **66.6** | **31** | |
| **23** | **gi\|674653945** | **GP41** | **96** | | **80** | | **4.13** | | **17.7** | **4** | | **-** | | **-** | **-** | |
| **24** | **gi\|674653951** | **IAP2** | **102** | | **71** | | **-** | | **-** | **-** | | **4.16** | | **9.7** | **2** | |
| **25** | **gi\|674653955** | **Desmoplakin** | **106** | | **66** | | **2.36** | | **4.4** | **3** | | **24.6** | | **21.1** | **13** | |
| **26** | **gi\|674653957** | **Mabr108** | **108** | | **75** | | **2** | | **12.4** | **1** | | **-** | | **-** | **-** | |
| **27** | **gi\|674653966** | **FP25K** | **117** | | **61** | | **2** | | **6.7** | **1** | | **-** | | **-** | **-** | |
| **28** | **gi\|674653970** | **ChaB2** | **120** | | **60** | | **-** | | **-** | **-** | | **2.12** | | **15.4** | **1** | |
| **29** | **gi\|674653971** | **ChaB1** | **121** | | **58-59** | | **-** | | **-** | **-** | | **9.29** | | **39.6** | **5** | |
| **30** | **gi\|674653977** | **Mabr127** | **127** | | **-** | | **4.6** | | **46.7** | **5** | | **4.15** | | **53.3** | **5** | |
| **31** | **gi\|674653978** | **Mabr128** | **128** | | **-** | | **9.25** | | **25** | **11** | | **9.87** | | **16.5** | **6** | |
| **32** | **gi\|674653982** | **BJDP** | **132** | | **51** | | **-** | | **-** | **-** | | **5.21** | | **19** | **5** | |
| **33** | **gi\|674653993** | **PP31** | **143** | | **36** | | **2.02** | | **8.1** | **1** | | **-** | | **-** | **-** | |
| **34** | **gi\|674653995** | **V-Ubi** | **145** | | **35** | | **-** | | **-** | **-** | | **11.2** | | **59** | **6** | |
| **35** | **gi\|674653999** | **LEF6** | **149** | | **28** | | **2.23** | | **7.1** | **1** | | **-** | | **-** | **-** | |
| **36** | **gi\|674654001** | **P26-2** | **151** | | **136** | | **2.95** | | **7.5** | **2** | | **7.77** | | **15.4** | **4** | |
| **37** | **gi\|674654008** | **ODV-EC27** | **158** | | **143** | | **2.03** | | **5.8** | **3** | | **9.04** | | **18** | **4** | |
| **38** | **gi\|674654009** | **ODV-E18** | **159** | | **144** | | **2** | | **11.8** | **2** | | **2** | | **11.8** | **1** | |
| **39** | **gi\|674654010** | **49K** | **160** | | **142** | | **4.09** | | **8.7** | **3** | | **2.08** | | **3** | **1** | |

**^a^ Score was given by ProteinPilot software. The Score value is calculated by the following formula: Score = -log(1-PercentConfidence/100). Protein identitied with Score higher than 1.3 (p<0.05) were considered significant and listed in this table.**

**^b^ The percentage of matching amino acids of identified peptides with confidence greater than 95% divided by the total number of amino acids in the sequence.**

**^c^ The number of matching peptides with confidence more than 95%.**
