# Supplementary material for: Proteomic Analysis of Mamestra Brassicae Nucleopolyhedrovirus Progeny Virions from Two Different Hosts
Source: PLoS One. 2016 Apr 8;11(4):e0153365. doi: 10.1371/journal.pone.0153365 (PMC4825930; doi:10.1371/journal.pone.0153365)
Supplement: S3 Table — (DOCX) [file pone.0153365.s003.docx]

**S3 Table. Summary of proteomic studies of baculoviral BVs and ODVs.**

|  | ***Alpha* Group I** | | | | | ***Alpha* Group II** | | | | | ***Betaba*** | ***Delta*** |  |
| --- | --- | --- | --- | --- | --- | --- | --- | --- | --- | --- | --- | --- | --- |
| **Protein** | **AgMNPV** | | **AcMNPV** | | **BmNPV ODV** | **ChchNPV ODV** | **HearNPV** | | **MabrNPV** | | **PrGV  ODV** | **CuniNPV ODV** | **Functions [Ref (s)]** |
|  | **ODV** | **BV** | **ODV** | **BV** |  |  | **ODV** | **BV** | **ODV** | **BV** |  |  |  |
| **P74** | **+** |  | **+** |  | **+** | **+** | **+** |  | **+** |  | **+** | **+** | **Essential for *per os* infectivity [1]** |
| **PIF1** | **+** |  | **+** |  | **+** | **+** | **+** |  | **+** |  | **+** | **+** | **Essential for *per os* infectivity [2]** |
| **PIF2** | **+** |  | **+** |  |  | **+** | **+** |  | **+** |  | **+** | **+** | **Essential for *per os* infectivity [2]** |
| **PIF3** | **+** |  | **+** |  |  | **+** | **+** |  | **+** |  | **+** | **+** | **Essential for *per os* infectivity [2]** |
| **PIF4** |  |  | **+** | **+** |  |  | **+** |  | **+** |  | **+** |  | **Essential for *per os* infectivity [3,4]** |
| **PIF5** | **+** | **+** | **+** | **+** |  | **+** | **+** | **+** | **+** |  | **+** | **+** | **Essential for *per os* infectivity [5,6]** |
| **PIF6** |  |  | **+** |  |  | **+** | **+** |  | **+** |  | **+** |  | **Essential for *per os* infectivity [7]** |
| **VP91** | **+** |  | **+** |  | **+** | **+** | **+** |  | **+** |  | **+** | **+** | **Involved in *per os* infectivity and essential for nucelocapsid assembly [8]** |
| **E66** | **+** |  | **+** |  | **+** | **+** | **+** |  | **++** |  | **++** |  | **Important for *per os* infectivity [9]** |
| **AC145 (ChtB)** |  |  | **+** | **+** |  | **+** |  |  | **++** |  |  |  | **Enhance *per os* infectivity [10]** |
| **AC150** |  |  | **+** | **+** |  |  |  |  |  |  |  |  | **Enhance *per os* infectivity [10,11]** |
| **VEF** |  |  |  |  |  |  |  |  |  |  |  |  | **Enhance *per os* infectivity** |
| **F protein** |  | **+** | **+** | **+** |  |  |  | **+** |  | **+** | **+** | **+** | **Essential for BV infection in Group II baculoviruses [12-14]** |
| **GP64** |  | **+** |  | **+** |  | **+** |  |  |  |  |  |  | **Essential for BV infection in Group I baculoviruses [15]** |
| **AC66** | **+** | **+** | **+** | **+** | **+** | **+** | **+** | **+** | **+** | **+** |  |  | **Important for nuclear egress of nucleocapsid and BV & ODV production but not essential for AcMNPV [16];** |
| **AC78** |  |  | **+** |  |  |  | **+** |  | **+** |  | **+** |  | **Essential for BV production and ODV embedding into the occlusion body [17,18]** |
| **AC81** |  |  |  |  |  | **+** |  |  |  |  |  | **+** | **Important for BV and ODV production but not essential [19]** |
| **E25** | **+** | **+** | **+** | **+** | **+** | **+** | **+** | **+** | **+** | **+** |  |  | **Essential for BV infectivity and ODV formation [20,21]** |
| **P33** | **+** | **+** | **+** | **+** |  | **+** | **+** |  | **+** |  | **+** | **+** | **Essential for efficient BV production [22,23] and multiply-ODV formation [22]** |
| **GP41** | **+** |  | **+** |  | **+** | **+** | **+** | **+** | **+** | **+** | **+** | **+** | **Required for nucleocapsid egress from nucleus [24] and essential for BV production [24,25]** |
| **49K** | **+** | **+** | **+** | **+** | **+** | **+** | **+** | **+** | **+** | **+** | **+** | **+** | **Essential for BV production and nucelocapsid maturation [26-28] and ODV envelopment [28]** |
| **EC43** | **+** | **+** | **+** | **+** | **+** | **+** | **+** | **+** | **+** |  | **+** | **+** | **Essential for nucleocapsid formation but not viral DNA replication [29,30]; involved in nucleocapsid transport and envelopment in infected cell [31,32]** |
| **P18** |  |  | **+** |  |  |  |  |  | **+** |  |  |  | **Essential for ODV envelopment and nuclear egress of nucleocapsids but not DNA replication [33]** |
| **P48** | **+** | **+** |  |  | **+** | **+** | **+** |  | **+** | **+** | **+** |  | **Essential for BV production[25,34] and ODV envelopment [34]** |
| **P12** |  |  | **+** |  |  | **+** | **+** |  | **+** | **+** | **+** |  | **Essential for mediating nuclear localization of G-Actin [35] and BV production [25,35]** |
| **P78/83** | **+** |  | **+** | **+** | **+** | **+** | **+** | **+** | **+** | **+** | **+** |  | **Essential for nucleocapsid assembly [36]; mediate actin-driven mobility of nucleaocapsid [37] and actin nucleation [38]** |
| **VP80** | **+** | **+** | **+** | **+** | **+** | **+** | **+** | **+** | **+** | **+** |  |  | **Essential for nucleocapsid transport from the periphery of the nucleus on their way to the plasma membrane to form BVs [39]** |
| **EXON0** |  |  | **+** |  |  |  |  |  |  |  |  |  | **Important for efficient nuclear egress of nucleocapsid and production of BV [40-43]** |
| **AC75** |  |  |  |  |  | **+** | **+** | **+** | **+** | **+** | **+** |  | **Essential [25,44] and required in nuclear egress of nucleocapsids [44]** |
| **E18** | **+** | **+** | **+** | **+** |  | **+** | **+** | **+** | **+** | **+** | **+** |  | **Essential for BV production but not for DNA replication [45]** |
| **EC27** | **+** | **+** | **+** | **+** |  | **+** | **+** | **+** | **+** | **+** | **+** | **+** | **Essential for BV production and nucleocaspsid formation [27]** |
| **VP39** | **+** | **+** | **+** | **+** | **+** | **+** | **+** | **+** | **+** | **+** | **+** | **+** | **Essential [25]; major capsid protein [46,47]** |
| **VP1054** | **+** | **+** | **+** | **+** |  |  | **+** | **+** | **+** |  | **+** | **+** | **Essential for nucleocapsid assembly [48]** |
| **38K** |  | **+** | **+** | **+** |  | **+** | **+** | **+** | **+** | **+** | **+** | **+** | **Essential for nucleocapsid assembly [49]** |
| **C42** | **+** | **+** | **+** | **+** |  | **+** | **+** | **+** | **+** | **+** | **+** |  | **Essential for nucleocapsid assembly [27] and recruiting P78/83 to nucleus [50], nuclear actin polymerization [51] and nucleocapsid morphogenesis [27]** |
| **AC53** |  |  |  |  |  |  | **+** |  | **+** |  |  |  | **Essential for nucleocapsid assembly and virus production but not DNA replication [52]** |
| **P6.9** | **+** |  | **+** | **+** |  | **+** | **+** | **+** | **+** | **+** | **+** | **+** | **Essential for of BV and ODV production [53]; nucleocapsid core DNA-binding protein [54-57]** |
| **VLF-1** | **+** | **+** | **+** | **+** |  | **+** | **+** | **+** | **+** |  | **+** | **+** | **Essential for capsid assembly [58-60] and maturation of DNA[59,60]** |
| **ALK-EXO** | **+** |  | **+** |  |  |  |  |  | **+** |  |  |  | **Essential [61,62]; may participate in the maturation of viral DNA or packaging of the DNA into virions [61,63]** |
| **BJDP** |  | **+** | **+** | **+** | **+** | **+** | **+** |  | **+** | **+** |  |  | **Essential for BV production [25]** |
| **AC73** |  |  | **+** | **+** |  |  |  |  |  |  |  |  | **Essential for BV production [25]** |
| **AC76** |  | **+** | **+** | **+** |  |  | **+** | **+** |  |  |  |  | **Involved in intranuclear microvesicle formation and essential for BV and ODV production [64]** |
| **AC78** |  |  |  |  |  | **+** |  |  |  |  |  |  | **Essential for BV production[25] and ODV embedding into the occlusion body [17,18]** |
| **AC106** |  |  |  |  |  |  |  |  |  | **+** |  |  | **Essential for BV production [25]** |
| **AC132** |  |  | **+** | **+** |  |  |  |  | **+** | **+** |  |  | **Essential for BV production [25,65]** |
| **LEF1** | **+** |  | **+** |  | **+** |  |  |  |  |  |  |  | **Essential for production of both BV and ODV [25,66]; has DNA primase activity [67]** |
| **LEF2** |  |  | **+** | **+** |  |  |  |  |  |  |  |  | **Essential for DNA replication [68-70]** |
| **DNA-pol** | **+** |  | **+** |  | **+** |  | **+** |  |  |  | **+** |  | **Essential for DNA replication [71]** |
| **Helicase** | **+** | **+** | **+** |  |  |  | **+** |  | **Helicase2** |  | **Helicase1** |  | **Essential [25]** |
| **PK1** | **+** |  |  | **+** | **PK2** |  |  |  |  |  |  |  | **Essential [72]** |
| **LEF3** | **+** | **+** | **+** | **+** |  |  | **+** |  | **+** |  | **+** |  | **Essential for DNA replication [73-76] and virion production[25]** |
| **IE1** | **+** |  | **+** |  |  |  |  |  |  | **+** |  |  | **Essential for baculovirus early replication [77]; DNA binding protein [78-81]** |
| **ME53** |  | **+** | **+** | **+** |  |  |  | **+** | **+** | **+** | **+** |  | **Involved in viral replication and important for efficient BV and ODV production [82,83]** |
| **LEF6** |  |  | **+** | **+** |  | **+** | **+** | **+** | **+** | **+** |  |  | **Important but not essential for production of BV and ODV [84]** |
| **LEF9** |  |  |  |  |  |  |  |  |  |  |  | **+** | **Essential [25];** |
| **PP31** | **+** | **+** |  | **+** |  |  |  |  |  |  |  |  | **Not essential [85]** |
| **LEF12** |  |  |  |  |  |  | **+** | **+** |  |  |  |  | **Not essential and deletion may have no or mild effect on BV and ODV production [25,86]** |
| **PTP** | **+** |  | **+** | **+** | **+** | **PTP2** |  |  | **PTP2** |  |  |  | **Not essential [72,87]** |
| **EGT** | **+** |  |  | **+** |  |  |  | **+** |  | **+** |  |  | **Not essential [72,88]** |
| **FGF** |  |  |  |  |  |  |  | **+** |  | **+** | **FGF3** |  | **Not essential [89]; stimulates host cell motility [89,90] and is involved in efficient BV production and systematic infection[89,91,92]** |
| **V-Ubi** |  | **+** |  | **+** |  | **+** | **+** | **+** | **+** | **+** |  |  | **Not essential [93]; may block destruction of viral protein(s) by the host degradative pathway [94] and confer a slight growth advantage under certain conditions [93]** |
| **SOD** |  |  | **+** | **+** |  | **+** | **+** |  | **+** |  |  |  | **Not essential [95]** |
| **PCNA** |  |  | **+** | **+** |  |  |  |  |  |  |  |  | **Not essential [96]; accelerates expression of late genes [97,98]** |
| **HCF-1** |  |  | **+** |  |  |  |  |  |  |  |  |  | **Not essential [99]; affects virus replicate rate in tissue-specific manner [99]** |
| **PNK/PNL** | **+** | **+** | **+** |  |  |  |  |  |  |  |  |  | **Not essential for viral replication [100]** |
| **IAPs** | **IAP1** | **IAP2** |  | **IAP2** |  | **IAP3** |  | **IAP2** | **IAP-2,**  **IAP3** | **IAP2** |  |  | **Not essential [101]; antiapotosis [102]；** |
| **E26** | **+** |  | **+** | **+** |  |  |  |  |  |  |  |  | **Not essential [103]** |
| **PAGR** |  |  |  |  |  | **+** | **+** | **+** | **+** | **+** |  |  | **Not essential [104]** |
| **HE65** |  |  |  |  |  |  |  |  |  | **+** |  |  | **Not essential and deletion may have no or mild effect on BV and ODV production [25,35]** |
| **FP25K** | **+** | **+** | **+** | **+** | **+** | **+** | **+** | **+** |  | **+** |  |  | **Not essential [105-108]; mutations result in reduced ODV production, increased BV production [109], and altered envelopment [110,111]; participates in ODV membrane protein transport into nucleus and interacts with host importin α-16 [112]** |
| **POLH** | **+** |  | **+** | **+** | **+** | **+** | **+** | **+** | **+** | **+** | **Granulin** | **+** | **Not essential for DNA and BV production [113], major polyhedron protein [113,114] responsible for ODV specific occlusion into polyhedra and pohydra formation [114,115]** |
| **PEP** | **+** | **+** | **+** | **+** |  | **+** | **+** | **+** | **+** | **+** | **++** |  | **Not essential for polyhedral potency and BV production [116]; polyhedron membrane protein [117,118]; deletion prevents larval liquefaction and lysis of cultured cell [116]** |
| **P10** | **+** |  | **+** |  |  |  | **+** | **+** | **+** |  | **+** |  | **Not essential [25] but involved in polyhedron formation [117,119-121]** |
| **GP16** |  |  |  |  |  | **+** |  |  |  |  |  |  | **Not essential [25]** |
| **GP37** |  |  |  | **+** |  |  |  | **+** |  | **+** |  |  | **Not essential [122]** |
| **V-ChiA** |  |  |  | **+** |  |  |  | **+** |  | **+** |  |  | **Crucial for V-CATH processing [123,124] and the liquefaction of infected larvae [123,125]** |
| **V-CATH** |  |  |  | **+** |  |  |  | **+** | **+** | **+** |  |  | **Crucial for the liquefaction of infected larvae [125]** |
| **CG30** | **+** |  | **+** |  |  |  |  |  |  |  |  |  | **Not essential [25,126-128]; brings BV a subtle growth advantage over deletion mutant and is involved in polyhedra production [25,126-128]** |
| **Telokin** |  |  | **+** |  |  |  |  |  | **+** |  |  |  | **Not essential [25,129]** |
| **AC58/59^§^** |  |  | **+** | **+** |  | **+** | **+** | **+** | **+** | **+** |  |  | **Not essential [25,130]; DNA-binding protein, ChaB homologue [131,132]** |
| **AC60**  **(ChaB)** |  |  | **+** |  |  | **+** | **+** | **+** | **+** | **+** |  |  | **Not essential and may have no or mild effect on BV and ODV production [25]; DNA-binding protein, ChaB homologue [131,132];** |
| **BROs** |  |  |  |  | **BRO-E** |  | **BRO-C** |  |  |  |  | **+** | **Important for BV production but may not essential [25,133]; DNA binding protein [134]** |
| **P24** | **+** | **+** |  | **+** |  |  | **+** | **+** | **+** |  |  |  | **Not essential [133,135,136]; affects pathogenesis in larvae specifically [133]** |
| **P26** |  |  |  |  |  |  |  | **+** | **P26-2** | **P26-2** |  |  | **Auxiliary for virus replication and transmission [137]; may act in concert with P74 and p10 to regulate the virion occlusion process [138]** |
| **P43** |  |  | **+** |  |  |  |  |  |  |  |  |  | **Not essential for either BV and ODV production [25,139]** |
| **AC4** |  |  |  |  |  |  |  |  | **++** | **+** |  |  | **Not essential and deletion may have no or mild effect onBV and ODV production [25,35]** |
| **AC5** |  |  | **+** |  |  |  |  |  |  |  |  |  | **Not essential and may have no or mild effect on BV production [25]** |
| **AC13** | **+** |  |  |  |  |  |  |  |  |  |  |  | **Not essential [25]** |
| **AC17** |  |  |  |  |  |  |  | **+** |  |  |  |  | **Not essential but involved in BV production and infectivity [25,103,140]** |
| **AC18** | **+** | **+** |  |  |  | **+** |  |  |  |  |  |  | **Not essential; deletion does not reduce AcMNPV infectivity in LD_50_ bioassay but results in delayed mortality in LT_50_ bioassay for *Trichoplusia ni* [141]** |
| **AC26** |  |  |  | **+** |  |  | **+** | **+** | **+** |  |  |  | **Not essential and deletion may have mild effect on BV and ODV production [25,142]** |
| **AC30** |  |  | **+** |  |  |  |  |  |  |  |  |  | **Not essential [143]; deletion results in delayed mortality of larvae [143]** |
| **AC34** |  |  |  |  |  | **+** |  |  | **+** |  |  |  | **Important for BV production [25,144];** |
| **AC38** |  |  |  |  |  |  |  |  | **+** | **+** |  |  | **Important [145]; has the activity of ADP-ribose pyrophosphatase and deletion results in significant reduction in BV production [145]** |
| **AC43** |  |  |  |  |  |  |  |  | **+** |  |  |  | **Not essential [25,146]; deletion does not affect BV production but results in reduction of OB production [25,146]** |
| **AC63** |  |  |  |  |  |  | **+** |  |  |  |  |  | **Not essential and deletion may have no or mild effect on BV and ODV production [25]** |
| **AC74** |  | **+** | **+** | **+** |  |  | **+** | **+** |  |  |  |  | **Not essential for viral replication and efficient BV production [147]** |
| **AC79** |  |  | **+** |  | **+** |  |  |  |  |  |  |  | **Important for BV production but not essential [25,148]** |
| **AC108** |  |  | **+** |  |  | **+** | **+** |  | **+** |  |  |  | **Not essential and deletion may have no or mild effect on BV and ODV production [25,149]** |
| **AC110** |  |  |  |  |  | **+** | **+** |  |  |  |  |  | **Not essential for BV production [35]** |
| **AC114** |  | **+** | **+** | **+** | **+** |  |  |  |  |  |  |  | **Not essential for BV and ODV production [25,150]** |
| **AC124** |  |  | **+** | **+** |  |  |  |  |  |  |  |  | **Not essential for BV production [35]** |
| **AC146 (EP23)** |  |  |  |  |  | **+** | **+** |  |  |  |  |  | **Essential for BV production [25,151]** |
| **AC151** |  |  |  |  |  |  |  |  | **++** |  |  |  | **Important for BV and ODV production [152] and maybe involved in cell cycle arrest[153] and DNA replication[70]** |
| **Hoar** |  |  |  |  |  |  | **+** |  |  |  |  |  | **Unknown** |
| **HA44** |  |  |  |  |  | **+** | **+** | **+** | **+** | **+** |  |  | **Unknown** |
| **HA45** |  |  |  |  |  | **+** | **+** |  | **+** | **+** |  |  | **Unknown** |
| **HA83** |  |  |  |  |  |  | **+** |  |  |  |  |  | **Unknown** |
| **Other specific proteins** |  | **AG112,**  **AG130** |  |  |  | **Chch105, Chch123, Chch 135** | **HA107,HA121,**  **HA122** | **PKIP,**  **HA57,**  **HA107,**  **HA129** | **RR1**  **RR2**  **NRK**  **EP23**  **Mabr12**  **Mabr17**  **Mabr21**  **Mabr23**  **Mabr52**  **Mabr56**  **Mabr58**  **Mabr64**  **Mabr83**  **Mabr100**  **Mabr110**  **Mabr112** | **Mabr56,** | **Pr29,Pr35,**  **Pr42,Pr54,**  **Pr83,Pr84,**  **Pr111,**  **Pr114, PEP/P10** | **22 other CuniNPV proteins** | **Unknown** |

**The proteomic results in this study were listed with other proteomic analysis of baculoviral virions, inlcuding AgMNPV BV and ODV [154], AcMNPV BV [155] and ODV [156], BmNPV ODV [157], ChchNPV ODV [158], HearNPV BV [159] and ODV [159,160], PrGV ODV [161] and CuniNPV ODV [162].**

**“+” indicates presence of viral protein in cirions and its number indicates number of viral protein homologues found in virions.**

**References**

1. Haas-Stapleton EJ, Washburn JO, Volkman LE (2004) P74 mediates specific binding of Autographa californica M nucleopolyhedrovirus occlusion-derived virus to primary cellular targets in the midgut epithelia of Heliothis virescens Larvae. J Virol 78: 6786-6791.

2. Ohkawa T, Washburn JO, Sitapara R, Sid E, Volkman LE (2005) Specific binding of Autographa californica M nucleopolyhedrovirus occlusion-derived virus to midgut cells of Heliothis virescens larvae is mediated by products of pif genes Ac119 and Ac022 but not by Ac115. J Virol 79: 15258-15264.

3. Huang H, Wang M, Deng F, Wang H, Hu Z (2012) ORF85 of HearNPV encodes the per os infectivity factor 4 (PIF4) and is essential for the formation of the PIF complex. Virology 427: 217-223.

4. Fang M, Nie Y, Harris S, Erlandson MA, Theilmann DA (2009) Autographa californica multiple nucleopolyhedrovirus core gene ac96 encodes a per Os infectivity factor (PIF-4). J Virol 83: 12569-12578.

5. Sparks WO, Harrison RL, Bonning BC (2011) Autographa californica multiple nucleopolyhedrovirus ODV-E56 is a per os infectivity factor, but is not essential for binding and fusion of occlusion-derived virus to the host midgut. Virology 409: 69-76.

6. Xiang X, Chen L, Guo A, Yu S, Yang R, et al. (2011) The Bombyx mori nucleopolyhedrovirus (BmNPV) ODV-E56 envelope protein is also a per os infectivity factor. Virus Res 155: 69-75.

7. Nie Y, Fang M, Erlandson MA, Theilmann DA (2012) Analysis of the Autographa californica Multiple Nucleopolyhedrovirus Overlapping Gene Pair lef3 and ac68 Reveals that AC68 Is a Per Os Infectivity Factor and that LEF3 Is Critical, but Not Essential, for Virus Replication. J Virol 86: 3985-3994.

8. Zhu S, Wang W, Wang Y, Yuan M, Yang K (2013) The baculovirus core gene ac83 is required for nucleocapsid assembly and per os infectivity of Autographa californica nucleopolyhedrovirus. J Virol 87: 10573-10586.

9. Xiang X, Chen L, Hu X, Yu S, Yang R, et al. (2011) Autographa californica multiple nucleopolyhedrovirus odv-e66 is an essential gene required for oral infectivity. Virus Res 158: 72-78.

10. Lapointe R, Popham HJ, Straschil U, Goulding D, O'Reilly DR, et al. (2004) Characterization of two Autographa californica nucleopolyhedrovirus proteins, Ac145 and Ac150, which affect oral infectivity in a host-dependent manner. J Virol 78: 6439-6448.

11. Zhang JH, Ohkawa T, Washburn JO, Volkman LE (2005) Effects of Ac150 on virulence and pathogenesis of Autographa californica multiple nucleopolyhedrovirus in noctuid hosts. J Gen Virol 86: 1619-1627.

12. Long G, Westenberg M, Wang H, Vlak JM, Hu Z (2006) Function, oligomerization and N-linked glycosylation of the Helicoverpa armigera single nucleopolyhedrovirus envelope fusion protein. J Gen Virol 87: 839-846.

13. Tan Y, Jiang L, Wang M, Yin F, Deng F, et al. (2008) Mutagenesis and nuclear magnetic resonance analyses of the fusion peptide of Helicoverpa armigera single nucleocapsid nucleopolyhedrovirus F protein. J Virol 82: 8138-8148.

14. Wang M, Yin F, Shen S, Tan Y, Deng F, et al. (2010) Partial functional rescue of Helicoverpa armigera single nucleocapsid nucleopolyhedrovirus infectivity by replacement of F protein with GP64 from Autographa californica multicapsid nucleopolyhedrovirus. J Virol 84: 11505-11514.

15. Monsma SA, Oomens AG, Blissard GW (1996) The GP64 envelope fusion protein is an essential baculovirus protein required for cell-to-cell transmission of infection. J Virol 70: 4607-4616.

16. Ke J, Wang J, Deng R, Wang X (2008) Autographa californica multiple nucleopolyhedrovirus ac66 is required for the efficient egress of nucleocapsids from the nucleus, general synthesis of preoccluded virions and occlusion body formation. Virology 374: 421-431.

17. Huang HC, Wang ML, Deng F, Hou DH, Arif BM, et al. (2014) The ha72 Core Gene of Baculovirus Is Essential for Budded Virus Production and Occlusion-Derived Virus Embedding, and Amino Acid K22 Plays an Important Role in Its Function. Journal of Virology 88: 705-709.

18. Tao XY, Choi JY, Kim WJ, Lee JH, Liu Q, et al. (2013) The Autographa californica Multiple Nucleopolyhedrovirus ORF78 Is Essential for Budded Virus Production and General Occlusion Body Formation. Journal of Virology 87: 8441-8450.

19. Ge JQ, Yang ZN, Tang XD, Xu HJ, Hong J, et al. (2008) Characterization of a nucleopolyhedrovirus with a deletion of the baculovirus core gene Bm67. J Gen Virol 89: 766-774.

20. Chen L, Hu X, Xiang X, Yu S, Yang R, et al. (2012) Autographa californica multiple nucleopolyhedrovirus odv-e25 (Ac94) is required for budded virus infectivity and occlusion-derived virus formation. Arch Virol 157: 617-625.

21. Chen L, Yang R, Hu X, Xiang X, Yu S, et al. (2013) The formation of occlusion-derived virus is affected by the expression level of ODV-E25. Virus Res 173: 404– 414.

22. Wu W, Passarelli AL (2010) Autographa californica multiple nucleopolyhedrovirus Ac92 (ORF92, P33) is required for budded virus production and multiply enveloped occlusion-derived virus formation. J Virol 84: 12351-12361.

23. Nie Y, Fang M, Theilmann DA (2011) Autographa californica multiple nucleopolyhedrovirus core gene ac92 (p33) is required for efficient budded virus production. Virology 409: 38-45.

24. Olszewski J, Miller LK (1997) A role for baculovirus GP41 in budded virus production. Virology 233: 292-301.

25. Ono C, Kamagata T, Taka H, Sahara K, Asano S, et al. (2012) Phenotypic grouping of 141 BmNPVs lacking viral gene sequences. Virus Res 165: 197-206.

26. Yang ZN, Xu HJ, Park EY, Zhang CX (2008) Characterization of Bombyx mori nucleopolyhedrovirus with a deletion of bm118. Virus Res 135: 220-229.

27. Vanarsdall AL, Pearson MN, Rohrmann GF (2007) Characterization of baculovirus constructs lacking either the Ac 101, Ac 142, or the Ac 144 open reading frame. Virology 367: 187-195.

28. McCarthy CB, Dai X, Donly C, Theilmann DA (2008) Autographa californica multiple nucleopolyhedrovirus ac142, a core gene that is essential for BV production and ODV envelopment. Virology 372: 325-339.

29. Lin L, Wang J, Deng R, Ke J, Wu H, et al. (2009) ac109 is required for the nucleocapsid assembly of Autographa californica multiple nucleopolyhedrovirus. Virus Res 144: 130-135.

30. Fang M, Nie Y, Theilmann DA (2009) Deletion of the AcMNPV core gene ac109 results in budded virions that are non-infectious. Virology 389: 66-74.

31. Alfonso V, Maroniche GA, Reca SR, Lopez MG, del Vas M, et al. (2012) AcMNPV Core Gene ac109 Is Required for Budded Virion Transport to the Nucleus and for Occlusion of Viral Progeny. Plos One 7: e46146.

32. Lehiy CJ, Wu W, Berretta MF, Passarelli AL (2013) Autographa californica M nucleopolyhedrovirus open reading frame 109 affects infectious budded virus production and nucleocapsid envelopment in the nucleus of cells. Virology 435: 442-452.

33. Yuan M, Huang Z, Wei D, Hu Z, Yang K, et al. (2011) Identification of Autographa californica Nucleopolyhedrovirus ac93 as a Core Gene and Its Requirement for Intranuclear Microvesicle Formation and Nuclear Egress of Nucleocapsids. J Virol 85: 11664-11674.

34. Yuan M, Wu W, Liu C, Wang Y, Hu Z, et al. (2008) A highly conserved baculovirus gene p48 (ac103) is essential for BV production and ODV envelopment. Virology 379: 87-96.

35. Gandhi KM, Ohkawa T, Welch MD, Volkman LE (2012) Nuclear localization of actin requires AC102 in Autographa californica multiple nucleopolyhedrovirus-infected cells. J Gen Virol 93: 1795-1803.

36. Wang Q, Wang Y, Liang C, Song J, Chen X (2008) Identification of a hydrophobic domain of HA2 essential to morphogenesis of Helicoverpa armigera nucleopolyhedrovirus. Journal of virology 82: 4072-4081.

37. Ohkawa T, Volkman LE, Welch MD (2010) Actin-based motility drives baculovirus transit to the nucleus and cell surface. J Cell Biol 190: 187-195.

38. Lanier LM, Volkman LE (1998) Actin binding and nucleation by Autographa california M nucleopolyhedrovirus. Virology 243: 167-177.

39. Marek M, Merten OW, Galibert L, Vlak JM, van Oers MM (2011) Baculovirus VP80 protein and the F-actin cytoskeleton interact and connect the viral replication factory with the nuclear periphery. J Virol 85: 5350-5362.

40. Fang M, Dai X, Theilmann DA (2007) Autographa californica multiple nucleopolyhedrovirus EXON0 (ORF141) is required for efficient egress of nucleocapsids from the nucleus. J Virol 81: 9859-9869.

41. Fang M, Nie Y, Dai X, Theilmann DA (2008) Identification of AcMNPV EXON0 (ac141) domains required for efficient production of budded virus, dimerization and association with BV/ODV-C42 and FP25. Virology 375: 265-276.

42. Fang M, Nie Y, Theilmann DA (2009) AcMNPV EXON0 (AC141) which is required for the efficient egress of budded virus nucleocapsids interacts with beta-tubulin. Virology 385: 496-504.

43. Dai X, Stewart TM, Pathakamuri JA, Li Q, Theilmann DA (2004) Autographa californica multiple nucleopolyhedrovirus exon0 (orf141), which encodes a RING finger protein, is required for efficient production of budded virus. J Virol 78: 9633-9644.

44. Shen H, Chen K, Yao Q, Zhou Y (2009) Characterization of the Bm61 of the Bombyx mori nucleopolyhedrovirus. Curr Microbiol 59: 65-70.

45. McCarthy CB, Theilmann DA (2008) AcMNPV ac143 (odv-e18) is essential for mediating budded virus production and is the 30th baculovirus core gene. Virology 375: 277-291.

46. Thiem SM, Miller LK (1989) Identification, sequence, and transcriptional mapping of the major capsid protein gene of the baculovirus Autographa californica nuclear polyhedrosis virus. J Virol 63: 2008-2018.

47. Pearson MN, Russell RL, Rohrmann GF, Beaudreau GS (1988) p39, a major baculovirus structural protein: immunocytochemical characterization and genetic location. Virology 167: 407-413.

48. Olszewski J, Miller LK (1997) Identification and characterization of a baculovirus structural protein, VP1054, required for nucleocapsid formation. J Virol 71: 5040-5050.

49. Wu W, Lin T, Pan L, Yu M, Li Z, et al. (2006) Autographa californica multiple nucleopolyhedrovirus nucleocapsid assembly is interrupted upon deletion of the 38K gene. J Virol 80: 11475-11485.

50. Wang Y, Wang Q, Liang C, Song J, Li N, et al. (2008) Autographa californica multiple nucleopolyhedrovirus nucleocapsid protein BV/ODV-C42 mediates the nuclear entry of P78/83. J Virol 82: 4554-4561.

51. Li K, Wang Y, Bai H, Wang Q, Song J, et al. (2010) The putative pocket protein binding site of Autographa californica nucleopolyhedrovirus BV/ODV-C42 is required for virus-induced nuclear actin polymerization. J Virol 84: 7857-7868.

52. Liu C, Li Z, Wu W, Li L, Yuan M, et al. (2008) Autographa californica multiple nucleopolyhedrovirus ac53 plays a role in nucleocapsid assembly. Virology 382: 59-68.

53. Wang M, Tuladhar E, Shen S, Wang H, van Oers MM, et al. (2010) Specificity of baculovirus P6.9 basic DNA-binding proteins and critical role of the C terminus in virion formation. J Virol 84: 8821-8828.

54. Tweeten KA, Bulla LA, Consigli RA (1980) Characterization of an Extremely Basic Protein Derived from Granulosis Virus Nucleocapsids. J Virol 33: 866-876.

55. Wilson ME, Mainprize TH, Friesen PD, Miller LK (1987) Location, transcription, and sequence of a baculovirus gene encoding a small arginine-rich polypeptide. J Virol 61: 661-666.

56. Wilson ME, Price KH (1988) Association of Autographa californica nuclear polyhedrosis virus (AcMNPV) with the nuclear matrix. Virology 167: 233-241.

57. Kelly DC, Brown DA, Ayres MD, Allen CJ, Walker IO (1983) Properties of the Major Nucleocapsid Protein of Heliothis-Zea Singly Enveloped Nuclear Polyhedrosis-Virus. Journal of General Virology 64: 399-408.

58. Li Y, Wang J, Deng R, Zhang Q, Yang K, et al. (2005) vlf-1 deletion brought AcMNPV to defect in nucleocapsid formation. Virus Genes 31: 275-284.

59. Vanarsdall AL, Okano K, Rohrmann GF (2006) Characterization of the role of very late expression factor 1 in baculovirus capsid structure and DNA processing. J Virol 80: 1724-1733.

60. Todd JW, Passarelli AL, Lu A, Miller LK (1996) Factors regulating baculovirus late and very late gene expression in transient-expression assays. J Virol 70: 2307-2317.

61. Okano K, Vanarsdall AL, Rohrmann GF (2004) Characterization of a baculovirus lacking the alkaline nuclease gene. J Virol 78: 10650-10656.

62. Li L, Rohrmann GF (2000) Characterization of a baculovirus alkaline nuclease. J Virol 74: 6401-6407.

63. Okano K, Vanarsdall AL, Rohrmann GF (2007) A baculovirus alkaline nuclease knockout construct produces fragmented DNA and aberrant capsids. Virology 359: 46-54.

64. Hu Z, Yuan M, Wu W, Liu C, Yang K, et al. (2010) Autographa californica multiple nucleopolyhedrovirus ac76 is involved in intranuclear microvesicle formation. Journal of virology 84: 7437-7447.

65. Yang M, Wang S, Yue XL, Li LL (2014) Autographa californica Multiple Nucleopolyhedrovirus orf132 Encodes a Nucleocapsid-Associated Protein Required for Budded-Virus and Multiply Enveloped Occlusion-Derived Virus Production. J Virol 88: 12586-12598.

66. Evans JT, Leisy DJ, Rohrmann GF (1997) Characterization of the interaction between the baculovirus replication factors LEF-1 and LEF-2. J Virol 71: 3114-3119.

67. Mikhailov VS, Rohrmann GF (2002) Baculovirus replication factor LEF-1 is a DNA primase. J Virol 76: 2287-2297.

68. Wu CP, Huang YJ, Wang JY, Wu YL, Lo HR, et al. (2010) Autographa californica multiple nucleopolyhedrovirus LEF-2 is a capsid protein required for amplification but not initiation of viral DNA replication. J Virol 84: 5015-5024.

69. Lu A, Miller LK (1995) The roles of eighteen baculovirus late expression factor genes in transcription and DNA replication. J Virol 69: 975-982.

70. Kool M, Ahrens CH, Goldbach RW, Rohrmann GF, Vlak JM (1994) Identification of genes involved in DNA replication of the Autographa californica baculovirus. Proc Natl Acad Sci U S A 91: 11212-11216.

71. Vanarsdall AL, Okano K, Rohrmann GF (2005) Characterization of the replication of a baculovirus mutant lacking the DNA polymerase gene. Virology 331: 175-180.

72. Katsuma S, Kawaoka S, Mita K, Shimada T (2008) Genome-wide survey for baculoviral host homologs using the Bombyx genome sequence. Insect Biochem Mol Biol 38: 1080-1086.

73. Li Y, Passarelli AL, Miller LK (1993) Identification, sequence, and transcriptional mapping of lef-3, a baculovirus gene involved in late and very late gene expression. J Virol 67: 5260-5268.

74. Hang X, Dong W, Guarino LA (1995) The lef-3 gene of Autographa californica nuclear polyhedrosis virus encodes a single-stranded DNA-binding protein. J Virol 69: 3924-3928.

75. Mikhailov VS, Okano K, Rohrmann GF (2005) The redox state of the baculovirus single-stranded DNA-binding protein LEF-3 regulates its DNA binding, unwinding, and annealing activities. J Biol Chem 280: 29444-29453.

76. Mikhailov VS, Okano K, Rohrmann GF (2006) Structural and functional analysis of the baculovirus single-stranded DNA-binding protein LEF-3. Virology 346: 469-478.

77. Schultz KL, Wetter JA, Fiore DC, Friesen PD (2009) Transactivator IE1 is required for baculovirus early replication events that trigger apoptosis in permissive and nonpermissive cells. J Virol 83: 262-272.

78. Leisy DJ, Rohrmann GF (2000) The Autographa californica nucleopolyhedrovirus IE-1 protein complex has two modes of specific DNA binding. Virology 274: 196-202.

79. Olson VA, Wetter JA, Friesen PD (2003) The highly conserved basic domain I of baculovirus IE1 is required for hr enhancer DNA binding and hr-dependent transactivation. J Virol 77: 5668-5677.

80. Ito E, Sahri D, Knippers R, Carstens EB (2004) Baculovirus proteins IE-1, LEF-3, and P143 interact with DNA in vivo: a formaldehyde cross-linking study. Virology 329: 337-347.

81. Nagamine T, Kawasaki Y, Iizuka T, Matsumoto S (2005) Focal distribution of baculovirus IE1 triggered by its binding to the hr DNA elements. J Virol 79: 39-46.

82. Xi Q, Wang J, Deng R, Wang X (2007) Characterization of AcMNPV with a deletion of me53 gene. Virus Genes 34: 223-232.

83. de Jong J, Arif BM, Theilmann DA, Krell PJ (2009) Autographa californica multiple nucleopolyhedrovirus me53 (ac140) is a nonessential gene required for efficient budded-virus production. J Virol 83: 7440-7448.

84. Lin G, Blissard GW (2002) Analysis of an Autographa californica multicapsid nucleopolyhedrovirus lef-6-null virus: LEF-6 is not essential for viral replication but appears to accelerate late gene transcription. J Virol 76: 5503-5514.

85. Yamagishi J, Burnett ED, Harwood SH, Blissard GW (2007) The AcMNPV pp31 gene is not essential for productive AcMNPV replication or late gene transcription but appears to increase levels of most viral transcripts. Virology 365: 34-47.

86. Guarino LA, Mistretta TA, Dong W (2002) Baculovirus lef-12 is not required for viral replication. J Virol 76: 12032-12043.

87. Kamita SG, Nagasaka K, Chua JW, Shimada T, Mita K, et al. (2005) A baculovirus-encoded protein tyrosine phosphatase gene induces enhanced locomotory activity in a lepidopteran host. Proc Natl Acad Sci U S A 102: 2584-2589.

88. Flipsen JT, Mans RM, Kleefsman AW, Knebel-Morsdorf D, Vlak JM (1995) Deletion of the baculovirus ecdysteroid UDP-glucosyltransferase gene induces early degeneration of Malpighian tubules in infected insects. Journal of virology 69: 4529-4532.

89. Detvisitsakun C, Cain EL, Passarelli AL (2007) The Autographa californica M nucleopolyhedrovirus fibroblast growth factor accelerates host mortality. Virology 365: 70-78.

90. Lehiy CJ, Martinez O, Passarelli AL (2009) Virion-associated viral fibroblast growth factor stimulates cell motility. Virology 395: 152-160.

91. Katsuma S, Horie S, Daimon T, Iwanaga M, Shimada T (2006) In vivo and in vitro analyses of a Bombyx mori nucleopolyhedrovirus mutant lacking functional vfgf. Virology 355: 62-70.

92. Means JC, Passarelli AL (2010) Viral fibroblast growth factor, matrix metalloproteases, and caspases are associated with enhancing systemic infection by baculoviruses. Proc Natl Acad Sci U S A 107: 9825-9830.

93. Reilly LM, Guarino LA (1996) The viral ubiquitin gene of Autographa californica nuclear polyhedrosis virus is not essential for viral replication. Virology 218: 243-247.

94. Haas AL, Katzung DJ, Reback PM, Guarino LA (1996) Functional characterization of the ubiquitin variant encoded by the baculovirus Autographa californica. Biochemistry 35: 5385-5394.

95. Tomalski MD, Eldridge R, Miller LK (1991) A baculovirus homolog of a Cu/Zn superoxide dismutase gene. Virology 184: 149-161.

96. Iwahori S, Ikeda M, Kobayashi M (2002) Generation and characterization of Autographa californica nucleopolyhedrovirus mutants defective in pcna gene homologue. Journal of Insect Biotechnology and Sericology 71: 129-139.

97. Crawford AM, Miller LK (1988) Characterization of an early gene accelerating expression of late genes of the baculovirus Autographa californica nuclear polyhedrosis virus. J Virol 62: 2773-2781.

98. Iwahori S, Ikeda M, Kobayashi M (2004) Association of Sf9 cell proliferating cell nuclear antigen with the DNA replication site of Autographa californica multicapsid nucleopolyhedrovirus. J Gen Virol 85: 2857-2862.

99. Lu A, Miller LK (1996) Species-specific effects of the hcf-1 gene on baculovirus virulence. J Virol 70: 5123-5130.

100. Durantel D, Croizier L, Ayres MD, Croizier G, Possee RD, et al. (1998) The pnk/pnl gene (ORF 86) of Autographa californica nucleopolyhedrovirus is a non-essential, immediate early gene. J Gen Virol 79 ( Pt 3): 629-637.

101. Griffiths CM, Barnett AL, Ayres MD, Windass J, King LA, et al. (1999) In vitro host range of Autographa californica nucleopolyhedrovirus recombinants lacking functional p35, iap1 or iap2. J Gen Virol 80 ( Pt 4): 1055-1066.

102. Clem RJ, Miller LK (1994) Control of programmed cell death by the baculovirus genes p35 and iap. Molecular and cellular biology 14: 5212-5222.

103. Nie Y, Theilmann DA (2010) Deletion of AcMNPV AC16 and AC17 results in delayed viral gene expression in budded virus infected cells but not transfected cells. Virology 404: 168-179.

104. Luo S, Zhang Y, Xu X, Westenberg M, Vlak JM, et al. (2011) Helicoverpa armigera nucleopolyhedrovirus occlusion-derived virus-associated protein, HA100, affects oral infectivity in vivo but not virus replication in vitro. J Gen Virol 92: 1324-1331.

105. Rosas-Acosta G, Braunagel SC, Summers MD (2001) Effects of deletion and overexpression of the Autographa californica nuclear polyhedrosis virus FP25K gene on synthesis of two occlusion-derived virus envelope proteins and their transport into virus-induced intranuclear membranes. J Virol 75: 10829-10842.

106. Katsuma S, Nakanishi T, Shimada T (2009) Bombyx mori nucleopolyhedrovirus FP25K is essential for maintaining a steady-state level of v-cath expression throughout the infection. Virus Res 140: 155-160.

107. Braunagel SC, Cox V, Summers MD (2009) Baculovirus data suggest a common but multifaceted pathway for sorting proteins to the inner nuclear membrane. J Virol 83: 1280-1288.

108. Kelly BJ, King LA, Possee RD, Chapple SD (2006) Dual mutations in the Autographa californica nucleopolyhedrovirus FP-25 and p35 genes result in plasma-membrane blebbing in Trichoplusia ni cells. J Gen Virol 87: 531-536.

109. Wu D, Deng F, Sun X, Wang H, Yuan L, et al. (2005) Functional analysis of FP25K of Helicoverpa armigera single nucleocapsid nucleopolyhedrovirus. J Gen Virol 86: 2439-2444.

110. Braunagel SC, Burks JK, Rosas-Acosta G, Harrison RL, Ma H, et al. (1999) Mutations within the Autographa californica nucleopolyhedrovirus FP25K gene decrease the accumulation of ODV-E66 and alter its intranuclear transport. J Virol 73: 8559-8570.

111. Harrison RL, Summers MD (1995) Mutations in the Autographa californica multinucleocapsid nuclear polyhedrosis virus 25 kDa protein gene result in reduced virion occlusion, altered intranuclear envelopment and enhanced virus production. J Gen Virol 76 ( Pt 6): 1451-1459.

112. Saksena S, Summers MD, Burks JK, Johnson AE, Braunagel SC (2006) Importin-alpha-16 is a translocon-associated protein involved in sorting membrane proteins to the nuclear envelope. Nat Struct Mol Biol 13: 500-508.

113. Rohrmann GF (1986) Polyhedrin structure. J Gen Virol 67 ( Pt 8): 1499-1513.

114. Ji X, Sutton G, Evans G, Axford D, Owen R, et al. (2010) How baculovirus polyhedra fit square pegs into round holes to robustly package viruses. Embo J 29: 505-514.

115. Lopez MG, Alfonso V, Carrillo E, Taboga O (2011) Description of a novel single mutation in the AcMNPV polyhedrin gene that results in abnormally large cubic polyhedra. Arch Virol 156: 695-699.

116. Bischoff DS, Slavicek JM (1999) Impact of deletion of the Lymantria dispar nucleopolyhedrovirus PEP gene on viral potency: Expression of the green fluorescent protein prevents larval liquefaction. Biological Control 14: 51-59.

117. Gross CH, Russell RL, Rohrmann GF (1994) Orgyia pseudotsugata baculovirus p10 and polyhedron envelope protein genes: analysis of their relative expression levels and role in polyhedron structure. J Gen Virol 75 ( Pt 5): 1115-1123.

118. Whitt MA, Manning JS (1988) A phosphorylated 34-kDa protein and a subpopulation of polyhedrin are thiol linked to the carbohydrate layer surrounding a baculovirus occlusion body. Virology 163: 33-42.

119. Carpentier DC, Griffiths CM, King LA (2008) The baculovirus P10 protein of Autographa californica nucleopolyhedrovirus forms two distinct cytoskeletal-like structures and associates with polyhedral occlusion bodies during infection. Virology 371: 278-291.

120. Lee SY, Poloumienko A, Belfry S, Qu X, Chen W, et al. (1996) A common pathway for p10 and calyx proteins in progressive stages of polyhedron envelope assembly in AcMNPV-infected Spodoptera frugiperda larvae. Arch Virol 141: 1247-1258.

121. van Oers MM, Flipsen JT, Reusken CB, Sliwinsky EL, Goldbach RW, et al. (1993) Functional domains of the p10 protein of Autographa californica nuclear polyhedrosis virus. J Gen Virol 74 ( Pt 4): 563-574.

122. Cheng X, Krell P, Arif B (2001) P34.8 (GP37) is not essential for baculovirus replication. J Gen Virol 82: 299-305.

123. Daimon T, Katsuma S, Shimada T (2007) Mutational analysis of active site residues of chitinase from Bombyx mori nucleopolyhedrovirus. Virus Res 124: 168-175.

124. Hom LG, Volkman LE (2000) Autographa californica M nucleopolyhedrovirus chiA is required for processing of V-CATH. Virology 277: 178-183.

125. Hawtin RE, Zarkowska T, Arnold K, Thomas CJ, Gooday GW, et al. (1997) Liquefaction of Autographa californica nucleopolyhedrovirus-infected insects is dependent on the integrity of virus-encoded chitinase and cathepsin genes. Virology 238: 243-253.

126. Passarelli AL, Miller LK (1994) In vivo and in vitro analyses of recombinant baculoviruses lacking a functional cg30 gene. J Virol 68: 1186-1190.

127. Zhang MJ, Cheng RL, Lou YH, Ye WL, Zhang T, et al. (2012) Disruption of Bombyx mori nucleopolyhedrovirus ORF71 (Bm71) results in inefficient budded virus production and decreased virulence in host larvae. Virus Genes 45: 161-168.

128. Ishihara G, Shimada T, Katsuma S (2013) Functional characterization of Bombyx mori nucleopolyhedrovirus CG30 protein. Virus Res 174: 52-59.

129. Iwanaga M, Kurihara M, Kobayashi M, Kang W (2002) Characterization of Bombyx mori nucleopolyhedrovirus orf68 gene that encodes a novel structural protein of budded virus. Virology 297: 39-47.

130. Zheng F, Huang Y, Long G, Sun X, Wang H (2011) Helicoverpa armigera single nucleocapsid nucleopolyhedrovirus ORF51 is a ChaB homologous gene involved in budded virus production and DNA replication. Virus Res 155: 203-212.

131. Li L, Li Z, Chen W, Liu C, Huang H, et al. (2006) Characterization of Spodoptera exigua multicapsid nucleopolyhedrovirus ORF100 and ORF101, two homologues of E. coli ChaB. Virus Res 121: 42-50.

132. Li Z, Li L, Yu H, Li S, Pang Y (2006) Characterization of two homologues of ChaB in Spodoptera litura multicapsid nucleopolyhedrovirus. Gene 372: 33-43.

133. Gomi S, Kamita SG, Maeda S (1999) Deletion analysis of all genes of Bombyx mori nucleopolyhedrovirus (BmNPV). RIKEN REVIEW: 39-41.

134. Zemskov EA, Kang W, Maeda S (2000) Evidence for nucleic acid binding ability and nucleosome association of Bombyx mori nucleopolyhedrovirus BRO proteins. J Virol 74: 6784-6789.

135. Schetter C, Oellig C, Doerfler W (1990) An insertion of insect cell DNA in the 81-map-unit segment of Autographa californica nuclear polyhedrosis virus DNA. J Virol 64: 1844-1850.

136. Gombart AF, Blissard GW, Rohrmann GF (1989) Characterization of the genetic organization of the HindIII M region of the multicapsid nuclear polyhedrosis virus of Orgyia pseudotsugata reveals major differences among baculoviruses. J Gen Virol 70 ( Pt 7): 1815-1828.

137. Simon O, Williams T, Caballero P, Possee RD (2008) Effects of Acp26 on in vitro and in vivo productivity, pathogenesis and virulence of Autographa californica multiple nucleopolyhedrovirus. Virus Res 136: 202-205.

138. Wang L, Salem TZ, Campbell DJ, Turney CM, Kumar CM, et al. (2009) Characterization of a virion occlusion-defective Autographa californica multiple nucleopolyhedrovirus mutant lacking the p26, p10 and p74 genes. J Gen Virol 90: 1641-1648.

139. Yu M, Carstens EB (2011) Characterization of an Autographa californica multiple nucleopolyhedrovirus mutant lacking the ac39(p43) gene. Virus Res 155: 300-306.

140. Yang ZN, Xu HJ, Thiem SM, Xu YP, Ge JQ, et al. (2009) Bombyx mori nucleopolyhedrovirus ORF9 is a gene involved in the budded virus production and infectivity. Journal of General Virology 90: 162-169.

141. Wang Y, Wu W, Li Z, Yuan M, Feng G, et al. (2007) ac18 is not essential for the propagation of Autographa californica multiple nucleopolyhedrovirus. Virology 367: 71-81.

142. Shen H, Zhou Y, Zhang W, Nin B, Wang H, et al. (2012) Characterization of Bombyx mori nucleopolyhedrovirus with a knockout of Bm17. Cytotechnology 64: 711-718.

143. Huang J, Hao B, Deng F, Sun X, Wang H, et al. (2008) Open reading frame Bm21 of Bombyx mori nucleopolyhedrovirus is not essential for virus replication in vitro, but its deletion extends the median survival time of infected larvae. J Gen Virol 89: 922-930.

144. Cai Y, Long Z, Qiu J, Yuan M, Li G, et al. (2012) An ac34 deletion mutant of Autographa californica nucleopolyhedrovirus exhibits delayed late gene expression and a lack of virulence in vivo. J Virol 86: 10432-10443.

145. Ge J, Wei Z, Huang Y, Yin J, Zhou Z, et al. (2007) AcMNPV ORF38 protein has the activity of ADP-ribose pyrophosphatase and is important for virus replication. Virology 361: 204-211.

146. Katsuma S, Shimada T (2009) Bombyx mori nucleopolyhedrovirus ORF34 is required for efficient transcription of late and very late genes. Virology 392: 230-237.

147. Guo ZJ, Qiu LH, An SH, Yao Q, Park EY, et al. (2010) Open reading frame 60 of the Bombyx mori nucleopolyhedrovirus plays a role in budded virus production. Virus Res 151: 185-191.

148. Wu W, Passarelli AL (2012) The Autographa californica M nucleopolyhedrovirus ac79 gene encodes an early gene product with structural similarities to UvrC and intron-encoded endonucleases that is required for efficient budded virus production. J Virol 86: 5614-5625.

149. Tang Q, Li G, Yao Q, Chen L, Lv P, et al. (2013) Bm91 is an envelope component of ODV but is dispensable for the propagation of Bombyx mori nucleopolyhedrovirus. J Invertebr Pathol 113: 70-77.

150. Wei W, Zhou Y, Lei C, Sun X (2012) Autographa californica multiple nucleopolyhedrovirus orf114 is not essential for virus replication in vitro, but its knockout reduces per os infectivity in vivo. Virus Genes 45: 360-369.

151. Dickison VL, Willis LG, Sokal NR, Theilmann DA (2012) Deletion of AcMNPV ac146 eliminates the production of budded virus. Virology 431: 29-39.

152. Prikhod'ko EA, Lu A, Wilson JA, Miller LK (1999) In vivo and in vitro analysis of baculovirus ie-2 mutants. J Virol 73: 2460-2468.

153. Prikhod'ko EA, Miller LK (1998) Role of baculovirus IE2 and its RING finger in cell cycle arrest. J Virol 72: 684-692.

154. Braconi CT, Ardisson-Araujo DM, Paes Leme AF, Oliveira JV, Pauletti BA, et al. (2014) Proteomic analyses of baculovirus Anticarsia gemmatalis multiple nucleopolyhedrovirus budded and occluded virus. J Gen Virol 95: 980-989.

155. Wang R, Deng F, Hou D, Zhao Y, Guo L, et al. (2010) Proteomics of the Autographa californica nucleopolyhedrovirus budded virions. J Virol 84: 7233-7242.

156. Braunagel SC, Russell WK, Rosas-Acosta G, Russell DH, Summers MD (2003) Determination of the protein composition of the occlusion-derived virus of Autographa californica nucleopolyhedrovirus. Proc Natl Acad Sci U S A 100: 9797-9802.

157. Liu X, Chen K, Cai K, Yao Q (2008) Determination of protein composition and host-derived proteins of Bombyx mori nucleopolyhedrovirus by 2-dimensional electrophoresis and mass spectrometry. Intervirology 51: 369-376.

158. Xu F, Ince IA, Boeren S, Vlak JM, van Oers MM (2011) Protein composition of the occlusion derived virus of Chrysodeixis chalcites nucleopolyhedrovirus. Virus Res 158: 1-7.

159. Hou D, Zhang L, Deng F, Fang W, Wang R, et al. (2013) Comparative proteomics reveal fundamental structural and functional differences between the two progeny phenotypes of a baculovirus. J Virol 87: 829-839.

160. Deng F, Wang R, Fang M, Jiang Y, Xu X, et al. (2007) Proteomics analysis of Helicoverpa armigera single nucleocapsid nucleopolyhedrovirus identified two new occlusion-derived virus-associated proteins, HA44 and HA100. J Virol 81: 9377-9385.

161. Wang XF, Zhang BQ, Xu HJ, Cui YJ, Xu YP, et al. (2011) ODV-associated proteins of the Pieris rapae granulovirus. J Proteome Res 10: 2817-2827.

162. Perera O, Green TB, Stevens SM, Jr., White S, Becnel JJ (2007) Proteins associated with Culex nigripalpus nucleopolyhedrovirus occluded virions. J Virol 81: 4585-4590.
