# Supplementary material for: Proteomic Analysis of Mamestra Brassicae Nucleopolyhedrovirus Progeny Virions from Two Different Hosts
Source: PLoS One. 2016 Apr 8;11(4):e0153365. doi: 10.1371/journal.pone.0153365 (PMC4825930; doi:10.1371/journal.pone.0153365)
Supplement: S4 Table — (DOCX) [file pone.0153365.s004.docx]

**S4 Table. Identification of host proteins associated with MabrNPV-CTa ODV.**

| **Category** | **Protein** | **Accession No.** | **Species** |  | **ODV from *H.armigera*** | | |  |  | **ODV from *S.exigua*** | | |  |
| --- | --- | --- | --- | --- | --- | --- | --- | --- | --- | --- | --- | --- | --- |
|  |  |  |  | **Score^a^** | | **% Cov^b^** | **Peptides^c^** | | **Score^a^** | | **% Cov^b^** | **Peptides^c^** | |
| **Cytoskeleton** | **Actin-4** | **gi\|525328769** | **Bombyx mori** | **16.32** | | **46.8** | **21** | | **10.96** | | **29.5** | **10** | |
|  | **Profilin** | **gi\|913315833** | **Amyelois transitella** | **6** | | **27.8** | **7** | | **6.04** | | **38.1** | **7** | |
|  | **Transgelin** | **gi\|509188677** | **Pararge aegeria** | **2.77** | | **19.6** | **3** | | **4.21** | | **14.4** | **2** | |
|  | **Twinstar** | **gi\|509182355** | **Pararge aegeria** | **4.93** | | **27.7** | **4** | |  | |  |  | |
| **Signaling** | **14-3-3 protein epsilon** | **gi\|910328986** | **Papilio xuthus** | **6.93** | | **21** | **4** | | **8** | | **17.9** | **4** | |
|  | **14-3-3 protein zeta** | **gi\|917957303** | **Papilio xuthus** | **10.06** | | **26.7** | **5** | | **6.76** | | **21.9** | **4** | |
| **Immunity** | **Cyclophilin A** | **gi\|298111994** | **Mythimna separata** | **8.56** | | **38.8** | **12** | | **8.81** | | **37.6** | **6** | |
| **Chaperone** | **Heat shock cognate 70 protein** | **gi\|728894870** | **Sesamia inferens** | **28.78** | | **29.4** | **28** | | **26.98** | | **27** | **20** | |
|  | **Heat shock protein 105** | **gi\|328670879** | **Helicoverpa armigera** |  | |  |  | | **2.01** | | **1.8** | **1** | |
| **Molecular transport** | **GTP-binding nuclear protein Ran** | **gi\|914615135** | **Papilio xuthus** | **5.6** | | **25.4** | **5** | | **2** | | **6.6** | **1** | |
| **Antioxidation** | **Thioredoxin** | **gi\|441481897** | **Helicoverpa armigera** | **2** | | **13.2** | **2** | | **2** | | **13.2** | **1** | |
| **Metabolism** | **ATP synthase subunit beta** | **gi\|914552935** | **Operophtera brumata** | **2.02** | | **9.6** | **3** | | **4.22** | | **18.1** | **8** | |
|  | **Glyceraldehyde-3-phosphate dehydrogenase** | **gi\|328670875** | **Helicoverpa armigera** | **9.81** | | **20.5** | **13** | | **6.21** | | **17.8** | **4** | |
|  | **Isocitrate dehydrogenase** | **gi\|768451008** | **Plutella xylostella** | **2.47** | | **6.9** | **2** | | **5.09** | | **10.8** | **3** | |
|  | **Peptidyl-prolyl cis-trans isomerase** | **gi\|913302155** | **Amyelois transitella** | **2** | | **18.8** | **8** | | **2.01** | | **6.7** | **1** | |
|  | **Succinate dehydrogenase** | **gi\|768448500** | **Plutella xylostella** | **2** | | **6** | **3** | | **4** | | **11.2** | **3** | |
| **Transcription and translation** | **Elongation factor-1 alpha** | **gi\|253509737** | **Temnora palpalis** | **4.86** | | **10.3** | **4** | | **3.09** | | **4.7** | **2** | |
|  | **Eukaryotic translation initiation factor 5A** | **gi\|914615861** | **Papilio xuthus** | **4** | | **16.3** | **2** | |  | |  |  | |
|  | **Translational controlled tumor protein** | **gi\|294862569** | **Helicoverpa armigera** | **4.8** | | **30.8** | **3** | | **2.02** | | **7** | **1** | |
|  | **SR-protein** | **gi\|348019723** | **Biston betularia** | **4.15** | | **16.6** | **3** | | **4.29** | | **21.1** | **3** | |
| **Vesicle transport** | **Annexin IX** | **gi\|328670889** | **Helicoverpa armigera** | **6.16** | | **18** | **3** | | **6.13** | | **13.9** | **3** | |
| **Unknown** | **Uuncharacterized protein LOC105395273** | **gi\|768447092** | **Plutella xylostella** | **10.51** | | **12.2** | **6** | | **42.81** | | **43.2** | **28** | |
|  | **Uncharacterized protein LOC106123913** | **gi\|914615420** | **Papilio xuthus** | **4.03** | | **27.6** | **3** | |  | |  |  | |

**^a^ Score was given by ProteinPilot software. The Score value is calculated by the following formula: Score = -log(1-PercentConfidence/100). Protein identitied with Score higher than 2.0 (p<0.01) were considered significant and listed in this table.**

**^b^ The percentage of matching amino acids of identified peptides with confidence greater than 95% divided by the total number of amino acids in the sequence.**

**^c^ The number of matching peptides with confidence more than 95%.**
