# Supplementary material for: Proteomic Analysis of Mamestra Brassicae Nucleopolyhedrovirus Progeny Virions from Two Different Hosts
Source: PLoS One. 2016 Apr 8;11(4):e0153365. doi: 10.1371/journal.pone.0153365 (PMC4825930; doi:10.1371/journal.pone.0153365)
Supplement: S5 Table — (DOCX) [file pone.0153365.s005.docx]

**S5 Table. Identification of host proteins associated with MabrNPV-CTa BV.**

| **Category** | **Protein** | **Accession No.** | **Species** |  | **BV from H.armigera** | | |  |  | **BV from S.exigua** | | |  |
| --- | --- | --- | --- | --- | --- | --- | --- | --- | --- | --- | --- | --- | --- |
|  |  |  |  | **Score^a^** | | **% Cov^b^** | **Peptides^c^** | | **Score^a^** | | **% Cov^b^** | **Peptides^c^** | |
| **Cytoskeleton** | **Actin-4** | **gi\|525328769** | **Bombyx mori** | **55.7** | | **90.2** | **109** | | **36.51** | | **65.4** | **31** | |
|  | **Alpha-tubulin** | **gi\|373427230** | **Helicoverpa armigera** | **5.57** | | **35.3** | **13** | | **10.86** | | **19.1** | **6** | |
|  | **Profilin** | **gi\|399227026** | **Spodoptera frugiperda** | **5.01** | | **27.8** | **4** | | **4.09** | | **27.8** | **2** | |
|  | **Transgelin** | **gi\|509188677** | **Pararge aegeria** |  | |  |  | | **5.37** | | **22.3** | **3** | |
|  | **Beta tubulin** | **gi\|333411420** | **Helicoverpa armigera** | **14.05** | | **32.4** | **10** | | **6.35** | | **11.9** | **4** | |
|  | **Twinstar** | **gi\|509182355** | **Pararge aegeria** | **17.82** | | **71** | **16** | | **6.56** | | **29.1** | **4** | |
| **Signaling** | **14-3-3 protein epsilon** | **gi\|910328986** | **Papilio xuthus** | **14.73** | | **43.1** | **12** | | **10.51** | | **32.4** | **7** | |
|  | **14-3-3 protein zeta** | **gi\|917957303** | **Papilio xuthus** | **10.04** | | **30.8** | **9** | | **8.42** | | **22.3** | **5** | |
|  | **Arginine kinase** | **gi\|356892492** | **Helicoverpa armigera** | **10.9** | | **24.8** | **8** | | **5.32** | | **21.3** | **3** | |
|  | **Ubiquitin** | **gi\|38373984** | **Helicoverpa armigera** | **2.84** | | **16.1** | **3** | | **3.64** | | **34.2** | **6** | |
|  | **C1A cysteine protease precursor** | **gi\|254746348** | **Spodoptera frugiperda** | **2** | | **1.7** | **1** | | **3.87** | | **4.6** | **3** | |
|  | **Cartilage oligomeric matrix protein** | **gi\|910319375** | **Papilio xuthus** | **2** | | **1.8** | **1** | | **6.3** | | **6.3** | **7** | |
|  | **Growth blocking peptide binding protein** | **gi\|341579602** | **Spodoptera exigua** | **2** | | **9** | **1** | | **2** | | **9.8** | **1** | |
|  | **Imaginal disc growth factor-like protein** | **gi\|85726208** | **Mamestra brassicae** | **13.07** | | **22.4** | **13** | | **23.62** | | **22.4** | **16** | |
|  | **Rab gdp-dissociation inhibitor** | **gi\|914556224** | **Operophtera brumata** | **2.85** | | **5.4** | **3** | | **2.17** | | **4.1** | **1** | |
|  | **Rab8** | **gi\|346987769** | **Helicoverpa armigera** | **2.84** | | **12.3** | **2** | |  | |  |  | |
| **Immunity** | **FK506-binding protein 2 precursor** | **gi\|822092498** | **Plutella xylostella** | **4.42** | | **17.2** | **4** | | **2** | | **5.7** | **1** | |
|  | **Hemocyte aggregation inhibitor protein precursor** | **gi\|357625069** | **Danaus plexippus** | **3.52** | | **20.5** | **13** | | **6** | | **22.8** | **13** | |
|  | **Hemocytin isoform X4** | **gi\|910353016** | **Papilio xuthus** | **11.15** | | **1.6** | **11** | | **19.34** | | **3.1** | **25** | |
|  | **Inter alpha-trypsin inhibitor** | **gi\|768441253** | **Plutella xylostella** | **3.55** | | **2.8** | **6** | | **2.91** | | **4.3** | **2** | |
|  | **Prophenoloxidase-1** | **gi\|159141810** | **Heliothis virescens** | **44.28** | | **42.5** | **68** | | **68.66** | | **60.7** | **49** | |
|  | **Serpin 3b** | **gi\|27733417** | **Manduca sexta** | **2.47** | | **5.9** | **2** | | **2** | | **4.4** | **3** | |
|  | **C-type lectin** | **gi\|106897087** | **Helicoverpa armigera** | **33.02** | | **69.6** | **45** | | **7.66** | | **6.1** | **21** | |
|  | **Serine protease inhibitor 3** | **gi\|557883331** | **Ostrinia furnacalis** | **2.21** | | **3** | **1** | | **2.28** | | **5.2** | **2** | |
|  | **Thymosin isoform 2** | **gi\|289900835** | **Helicoverpa armigera** | **5.92** | | **30.5** | **3** | | **4.01** | | **19.1** | **2** | |
|  | **C-type lectin 6** | **gi\|385202651** | **Helicoverpa armigera** | **5.3** | | **39.9** | **18** | | **8** | | **13.1** | **4** | |
| **Chaperone** | **Calreticulin precursor** | **gi\|914096634** | **Papilio polytes** | **5.37** | | **17.8** | **14** | | **6** | | **11.3** | **3** | |
|  | **Heat shock cognate 70** | **gi\|728894870** | **Sesamia inferens** | **20.67** | | **26.6** | **22** | | **23.85** | | **21.3** | **12** | |
|  | **Heat shock protein 90** | **gi\|379046536** | **Spodoptera litura** | **16.31** | | **13.4** | **19** | | **8.22** | | **7** | **4** | |
| **Molecular transport** | **Apolipophorin-III** | **gi\|300953022** | **Spodoptera exigua** | **2.11** | | **47.8** | **5** | | **16.48** | | **52.1** | **20** | |
|  | **Very high density lipoprotein** | **gi\|146345325** | **Helicoverpa zea** | **25.79** | | **12** | **17** | | **15.17** | | **9.4** | **26** | |
|  | **32 kDa ferritin subunit** | **gi\|17901818** | **Galleria mellonella** | **4.33** | | **20.3** | **6** | | **3.34** | | **24.6** | **10** | |
| **Antioxidation** | **Glutathione S-transferase** | **gi\|289719016** | **Helicoverpa armigera** | **8.3** | | **35** | **7** | | **6.38** | | **23** | **4** | |
|  | **Thioredoxin peroxidase** | **gi\|159459926** | **Helicoverpa armigera** | **2** | | **13.8** | **2** | | **2.01** | | **5.1** | **1** | |
|  | **Thioredoxin** | **gi\|441481897** | **Helicoverpa armigera** | **2** | | **13.2** | **1** | | **2** | | **13.2** | **1** | |
| **Metabolism** | **Glucose dehydrogenase** | **gi\|829569655** | **FAD, quinone** | **2** | | **3.8** | **2** | | **2.01** | | **4.5** | **3** | |
|  | **Glyceraldehyde-3-phosphate dehydrogenase** | **gi\|328670875** | **Helicoverpa armigera** | **7.39** | | **31.3** | **8** | | **6** | | **18.7** | **3** | |
|  | **Glycogen synthase** | **gi\|291278040** | **Spodoptera exigua** | **9.73** | | **15** | **6** | | **30.32** | | **26.9** | **16** | |
|  | **Isocitrate dehydrogenase** | **gi\|509160295** | **Pararge aegeria** | **5.36** | | **10.1** | **4** | | **2** | | **3.7** | **1** | |
|  | **P102 protein** | **gi\|334683151** | **Heliothis virescens** | **7.35** | | **23.1** | **8** | | **3.43** | | **13.2** | **4** | |
|  | **Alpha-amylase 2-like** | **gi\|827562702** | **Bombyx mori** | **8** | | **8.2** | **6** | | **8** | | **6.1** | **8** | |
|  | **Follicular epithelium yolk protein subunit** | **gi\|357606183** | **Danaus plexippus** | **2.53** | | **3.4** | **2** | | **2** | | **3.5** | **1** | |
|  | **Guanine nucleotide-binding protein subunit beta-1** | **gi\|913330119** | **Amyelois transitella** | **6.15** | | **18.8** | **8** | | **3.77** | | **6.2** | **2** | |
|  | **Midgut class 2 aminopeptidase N** | **gi\|37788338** | **Spodoptera exigua** | **11.47** | | **11.9** | **11** | | **5.21** | | **4.5** | **3** | |
|  | **Midgut class 3 aminopeptidase N** | **gi\|60739179** | **Spodoptera exigua** | **15.29** | | **14.2** | **12** | | **9.15** | | **5** | **4** | |
|  | **Midgut class 4 aminopeptidase N** | **gi\|37788344** | **Spodoptera exigua** | **11.08** | | **10.1** | **16** | | **11.82** | | **7.2** | **7** | |
| **DNA binding** | **Histone H4** | **gi\|914568958** | **Operophtera brumata** | **2** | | **32.5** | **7** | | **2** | | **20.9** | **4** | |
| **Transcription and Translation** | **40S ribosomal protein S14** | **gi\|914615589** | **Papilio xuthus** |  | |  |  | | **4** | | **15.9** | **2** | |
|  | **40S ribosomal protein S3** | **gi\|917957838** | **Papilio xuthus** | **9** | | **30.3** | **6** | | **2.1** | | **11.1** | **2** | |
|  | **40S ribosomal protein S8** | **gi\|54039568** | **SPOFR** | **9.99** | | **34.6** | **7** | | **6.16** | | **17.3** | **3** | |
|  | **60S acidic ribosomal protein P0** | **gi\|18253041** | **Spodoptera frugiperda** | **14.89** | | **35.2** | **11** | | **4.34** | | **7** | **2** | |
|  | **60S acidic ribosomal protein P2** | **gi\|18253045** | **Spodoptera frugiperda** | **4.17** | | **59.8** | **7** | | **8.66** | | **56.3** | **6** | |
|  | **60S ribosomal protein L10a** | **gi\|22001886** | **SPOFR** | **10.59** | | **20.3** | **12** | | **2.31** | | **6** | **1** | |
|  | **60S ribosomal protein L11** | **gi\|914614930** | **Papilio xuthus** | **3.13** | | **12.4** | **3** | | **5.26** | | **16.9** | **3** | |
|  | **60S ribosomal protein L13a** | **gi\|913335319** | **Amyelois transitella** | **5.74** | | **16.2** | **4** | |  | |  |  | |
|  | **60S ribosomal protein L15** | **gi\|27462516** | **Spodoptera frugiperda** | **4.8** | | **23** | **5** | | **8** | | **21.6** | **5** | |
|  | **60S ribosomal protein L17** | **gi\|915409921** | **Papilio xuthus** | **3.69** | | **5.9** | **2** | |  | |  |  | |
|  | **60S ribosomal protein L18** | **gi\|74938646** | **SPOFR** | **5.3** | | **23.5** | **5** | | **7.88** | | **27.3** | **4** | |
|  | **60S ribosomal protein L23** | **gi\|914615745** | **Papilio xuthus** | **4** | | **22.1** | **2** | | **6** | | **32.9** | **3** | |
|  | **60S ribosomal protein L31** | **gi\|914615789** | **Papilio xuthus** | **2.23** | | **11.3** | **2** | |  | |  |  | |
|  | **Elongation factor 1-alpha** | **gi\|918464054** | **Spodoptera frugiperda** | **18.45** | | **42.4** | **20** | | **10.72** | | **19** | **7** | |
|  | **Ribosomal protein L13** | **gi\|56463257** | **Helicoverpa zea** | **3.21** | | **9.5** | **2** | | **6.05** | | **14.9** | **3** | |
|  | **Ribosomal protein L21** | **gi\|54609233** | **Bombyx mori** | **2.13** | | **10.1** | **1** | | **2.03** | | **5** | **1** | |
|  | **Ribosomal protein L22** | **gi\|15213770** | **Spodoptera frugiperda** | **3.14** | | **24.5** | **6** | | **2.04** | | **7.5** | **1** | |
|  | **Ribosomal protein L3** | **gi\|18253047** | **Spodoptera frugiperda** | **2.86** | | **7** | **3** | | **2** | | **9.2** | **3** | |
|  | **Ribosomal protein L30** | **gi\|914575747** | **Operophtera brumata** | **2.44** | | **10.8** | **1** | | **4.97** | | **27.9** | **3** | |
|  | **Ribosomal protein L4** | **gi\|170280411** | **Heliothis virescens** | **13.95** | | **21.6** | **9** | | **8.28** | | **16.4** | **5** | |
|  | **Ribosomal protein L6e** | **gi\|161015753** | **Spodoptera exigua** | **2.55** | | **6.3** | **2** | | **4.89** | | **9.2** | **3** | |
|  | **Ribosomal protein L7** | **gi\|54609201** | **Bombyx mori** | **9.21** | | **20.1** | **6** | | **4.99** | | **13.7** | **3** | |
|  | **Ribosomal protein L7A** | **gi\|268306466** | **Manduca sexta** | **2.94** | | **9** | **1** | | **5.11** | | **11.2** | **4** | |
|  | **PDP protein** | **gi\|95103154** | **Bombyx mori** | **3.55** | | **13.5** | **2** | | **5.06** | | **18** | **3** | |
| **Protein posttranslational modification and degradation** | **Protein disulfide-isomerase** | **gi\|914572922** | **Operophtera brumata** | **4.48** | | **21.8** | **18** | | **13.9** | | **25.9** | **16** | |
|  | **Serine proteinase-like protein 1** | **gi\|208972549** | **Helicoverpa armigera** | **47.05** | | **63.3** | **66** | | **21.45** | | **36.2** | **20** | |
| **Vesicle transport** | **Vesicle amine transport protein** | **gi\|95103100** | **Bombyx mori** | **4.29** | | **5.4** | **2** | | **3.01** | | **5.1** | **2** | |
| **Unknown** | **Unknown protein product** | **gi\|156968297** | **Helicoverpa armigera** | **9.92** | | **26.1** | **10** | | **2** | | **7** | **2** | |
|  | **REPAT32** | **gi\|383932019** | **Spodoptera littoralis** | **8.6** | | **54.6** | **9** | | **8** | | **52.7** | **4** | |

**^a^ Score was given by ProteinPilot software. The Score value is calculated by the following formula: Score = -log(1-PercentConfidence/100). Protein identitied with Score higher than 2.0 (p<0.01) were considered significant and listed in this table.**

**^b^ The percentage of matching amino acids of identified peptides with confidence greater than 95% divided by the total number of amino acids in the sequence.**

**^c^ The number of matching peptides with confidence more than 95%.**
